# Total and Potentially Active Bacterial Communities Entrapped in a Late Glacial Through Holocene Ice Core From Scarisoara Ice Cave, Romania

**DOI:** 10.3389/fmicb.2019.01193

**Published:** 2019-05-29

**Authors:** Victoria I. Paun, Gonzalo Icaza, Paris Lavin, Constantin Marin, Alin Tudorache, Aurel Perşoiu, Cristina Dorador, Cristina Purcarea

**Affiliations:** ^1^Department of Microbiology, Institute of Biology, Bucharest, Romania; ^2^Laboratorio de Complejidad Microbiana y Ecología Funcional, Instituto Antofagasta, Universidad de Antofagasta, Antofagasta, Chile; ^3^Centre for Biotechnology and Bioengineering, Universidad de Antofagasta, Antofagasta, Chile; ^4^Departamento de Biotecnología, Facultad de Ciencias del Mar y Recursos Biológicos, Universidad de Antofagasta, Antofagasta, Chile; ^5^Laboratory of Hydrogeochemistry, “Emil Racovita” Institute of Speleology, Bucharest, Romania; ^6^“Emil Racovita” Institute of Speleology, Cluj-Napoca, Romania; ^7^Stefan cel Mare University of Suceava, Suceava, Romania

**Keywords:** bacterial diversity, geochemical composition, Illumina sequencing, perennial cave ice, potentially active microbiome

## Abstract

Our understanding of the icy-habitat microbiome is likely limited by a lack of reliable data on microorganisms inhabiting underground ice that has accumulated inside caves. To characterize how environmental variation impacts cave ice microbial community structure, we determined the composition of total and potentially active bacterial communities along a 13,000-year-old ice core from Scarisoara cave (Romania) through 16S rRNA gene Illumina sequencing. An average of 2,546 prokaryotic gDNA operational taxonomic units (OTUs) and 585 cDNA OTUs were identified across the perennial cave ice block and analyzed in relation to the geochemical composition of ice layers. The total microbial community and the putative active fraction displayed dissimilar taxa profiles. The ice-contained microbiome was dominated by Actinobacteria with a variable representation of Proteobacteria, while the putative active microbial community was equally shared between Proteobacteria and Firmicutes. Accordingly, a major presence of *Cryobacterium, Lysinomonas, Pedobacter*, and *Aeromicrobium* phylotypes homologous to psychrotrophic and psychrophilic bacteria from various cold environments were noted in the total community, while the prevalent putative active bacteria belonged to *Clostridium, Pseudomonas, Janthinobacterium, Stenotrophomonas*, and *Massilia* genera. Variation in the microbial cell density of ice strata with the dissolved organic carbon (DOC) content and the strong correlation of DOC and silicon concentrations revealed a major impact of depositional processes on microbial abundance throughout the ice block. Post-depositional processes appeared to occur mostly during the 4,000–7,000 years BP interval. A major bacterial composition shift was observed in 4,500–5,000-year-old ice, leading to a high representation of Beta- and Deltaproteobacteria in the potentially active community in response to the increased concentrations of DOC and major chemical elements. Estimated metabolic rates suggested the presence of a viable microbial community within the cave ice block, characterized by a maintenance metabolism in most strata and growth capacity in those ice deposits with high microbial abundance and DOC content. This first survey of microbial distribution in perennial cave ice formed since the Last Glacial period revealed a complex potentially active community, highlighting major shifts in community composition associated with geochemical changes that took place during climatic events that occurred about 5,000 years ago, with putative formation of photosynthetic biofilms.

## Introduction

The Earth’s cold biosphere covers a large variety of frozen habitats and supports several unique microbiomes (Priscu and Christner, 2004; Anesio and Laybourn-Parry, 2012; Gunde-Cimerman et al., 2012). The diversity and functional characteristics of bacterial communities from glaciers and polar ice-sheets (Miteva et al., 2009; Sheik et al., 2015), snow (Carpenter et al., 2000; Liu et al., 2009; Lopatina et al., 2016), polar soil, and permafrost (Zhang et al., 2007; McCann et al., 2016), mountain glacier forefields (Lapanje et al., 2012; Mateos-Rivera et al., 2016), sea-ice (Deming, 2002), Arctic and Antarctic frozen lakes (Priscu et al., 1998; Adams et al., 2014) have been extensively studied. In recent decades, complex microbial communities have been identified from Antarctic and Arctic ice sheets formed 157,000 years before present (Knowlton et al., 2013). Active microbiomes were discovered in ice and from other cold environments (Rivkina et al., 2000; Hansen et al., 2007; Dieser et al., 2010). Metabolic rates estimated for these active microbes were dependent on substrate temperature (Price and Sowers, 2004). A series of studies highlighted the potential importance of the geochemistry of ice substrates in shaping the abundance and composition of ice-contained microbiomes from several glacial habitats (Priscu et al., 1999; Skidmore et al., 2005; Liu et al., 2015) extending from the Last Glacial Maximum (Santibáñez et al., 2018).

Beyond these developments, little is known so far on the diversity and activity of microbial communities present in perennial ice accumulated in caves (Purcarea, 2018). These conserved habitats represent isolated and light-deprived frozen niches characterized by a low nutrient content and constant low temperatures (Sattler et al., 2002; Margesin et al., 2004). The presence of bacteria and fungi in the ice deposits from ice caves was first mentioned by Margesin et al. (2003, 2004). Diatom communities from ice deposits were reported from Canadian caves (Lauriol et al., 2006). Moreover, several cold-active autotrophic bacterial strains were isolated from the ice-rock interface of South Ice Cave, Oregon, United States (Popa et al., 2012). More recent studies have characterized prokaryotic (Tebo et al., 2015) and fungal (Connell and Staudigel, 2013) communities from sediments of three volcanic caves formed on Mount Erebus, Antarctica, and from sediments and ice deposits of Hawaii lava tubes (Teehera et al., 2017). To date, the highly preserved perennial cave ice accumulations represent understudied but potentially dynamic and robust archives for investigating the impact of climate and anthropogenic pollution on the diversity and viability of the ice entrapped microbiome. These habitats also represent a relatively novel source of cold-adapted microbial strains.

Scarisoara Ice Cave (Romania), the most extensively studied ice cave (Perşoiu et al., 2017), hosts the world’s oldest and largest perennial ice block (Holmlund et al., 2005). Over the last century, study of this underground ice block disentangled the climatic and glaciological associated processes in this cave (Racovita and Onac, 2000; Perşoiu et al., 2011b) and resulted in the successful reconstruction of climatic and environmental changes in the region (Onac et al., 2007; Feurdean et al., 2011; Perşoiu and Pazdur, 2011; Perşoiu et al., 2017). An early report revealed the presence of nitrifying bacteria in limestone sediments from this cave (Pop, 1949). The ice microbiome of Scarisoara cave was first studied during the last decade, revealing the presence of microorganisms in recently formed ice stalagmites (Hillebrand-Voiculescu et al., 2013). Cultured and uncultured microbial communities were identified in the subterranean ice block of this cave (Hillebrand-Voiculescu et al., 2014). The first chronological distribution of cultured bacteria in up to 900-year-old cave ice deposits (Itcus et al., 2016) was also investigated. Recently, the first high-throughput sequencing of a prokaryotic cave community characterized the uncultured prokaryotic community from Scarisoara ice strata formed during the last millennium using 454 pyrosequencing (Itcus et al., 2018). Additionally, the diversity of cultured fungi from this habitat was determined by denaturing gradient gel electrophoresis (DGGE) profiling (Brad et al., 2018). Moreover, the ice-contained fungal community composition in different ice strata up to 1,500-year-old across the cave ice block was recently assessed based on ITS2 Illumina sequencing (Mondini et al., 2019). These data revealed complex and culturable prokaryotic and eukaryotic microbial communities embedded in cave ice deposits, mostly accumulated during the last millennium, with putative variable response to temporal and environmental changes during ice formation. However, the dissimilarity of the microbial diversity inside the cave ice block at an extended temporal scale, and the environmental mechanisms shaping the ice microbiome abundance and composition in this type of glacial habitat are still unknown.

In this context, the current investigation represents a comprehensive study of the cave ice microbiome by unraveling the total and potentially active bacterial community structure in a ∼13,000-year-old ice core taken from Scarisoara Ice Cave, based on 16S rRNA gene Illumina sequencing, in relation with the age and geochemical composition of the ice strata. This first report on bacterial temporal distribution in cave ice deposited since the Late Glacial period provides an opportunity to advance our understanding of microbial community resilience and their ecological role in this type of habitat, thereby providing new leads for identification of microbial climatic proxies.

## Materials and Methods

### Study Site, Sampling and Ice Core Samples

**FIGURE 1:**
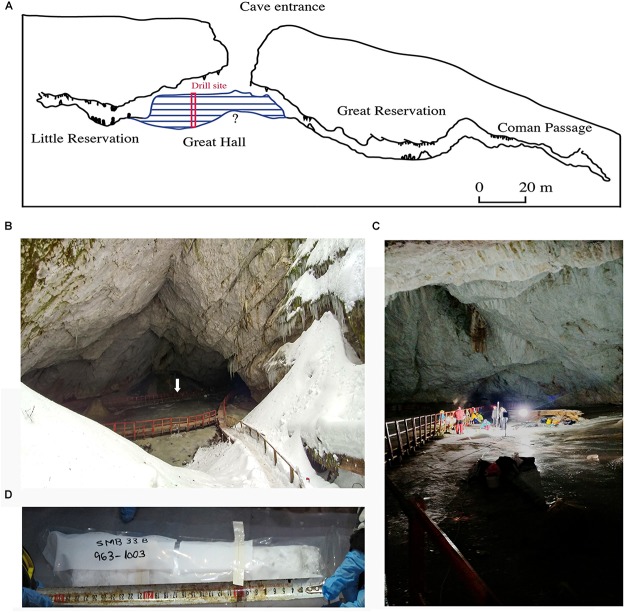
Scarisoara Ice Cave sampling location **(A)** Cave map profile indicating the position of the ice block (blue) of unknown (?) maximum depth and the drilling site. **(B)** Cave entrance and Great Hall area, indicating the ice block surface (arrow) (photo Paun V.I.). **(C)** Sampling site (photo Paun V.I.). **(D)** Ice core fragment placed in sterile plastic bag, corresponding to 963–1003 cm depth (photo Hillebrand-Voiculescu A).

Scarisoara Ice Cave (700 m long, 105 m deep, Figure 1A) is located in the Bihor Mountains, part of the Western Carpathian range (46°29′23″N, 22°48′35″E, 1,165 m above sea level). The cave harbors a 100,000 m^3^ perennial ice block previously carbon dated to >10,500 years (Holmlund et al., 2005; Hubbard, 2017; Perşoiu et al., 2017), and with an extrapolated age up to 13,000 calibrated years before present (cal BP), where “present” is defined as “Anno Domini (AD) 1950.” The descending morphology of the cave preserves negative temperatures throughout the year (Perşoiu et al., 2011a), leading to a continuous accumulation of perennial ice (Figure 1B). The ice block resulted from annual freezing of the supraglacial pond accumulated on top of the cave glacier, originating from dripping water infiltrations, rain and snowmelt water. Each layer is composed of 1–15 cm thick clear ice alternating with organic and inorganic sediment-rich strata (Feurdean et al., 2011; Perşoiu et al., 2011b).

Ice sampling was carried out in the Great Hall area of the cave (Figure 1C) by vertically drilling into the perennial ice block down to a depth of 25.33 m using a modified PICO electric drill (Koci and Kuivinen, 1984) manufactured by Heavy Duties S.R.L (Cluj-Napoca, Romania). To ensure aseptic collecting conditions, the auger and drill bits were sprayed with laboratory grade alcohol and flame sterilized for 5 s before each sampling. The ice surface around the drilling hole was also sterilized before each collecting step to avoid contamination. Ninety-seven ice core fragments (Figure 1D) of variable lengths (10–60 cm) were recovered and transferred to sterile plastic bags or wrapped in sterilized aluminum foils in the presence of an open flame. Ice samples were transported to the laboratory in thermal insulated 90 L containers under permanent frozen conditions and stored at -20°C until processing.

The chronology of the ice core was identified based on 26 ^14^C AMS ages (Perşoiu et al., 2017), and the depth-age model was constructed using a Bayesian model for the 0–22.5 m depth and linear extrapolation up to 25.33 m (Supplementary Figure S1). The calculated values corresponded to an approximate maximum age of 13000 years at the bottom of the core (25.33 m).

Fifteen ice samples were selected from various depths of the ice core (Table 1) for extraction of nucleic acids and Illumina sequencing. Each depth corresponded to an age interval of ∼300 years for the first millennium (samples SC100, SC400, and SC700), and ∼1,000 years for samples from SC1K to SC13K (Supplementary Figure S1).

**Table 1:** Ice core samples for selected gDNA (SC) and cDNA (SCR) Illumina sequencing.

BioSample code	Ice core depth (cm)	Age (years BP)
SC(R) 100	0–17.5	92 ± 25
SC(R) 400	167–204	430 ± 14
SC(R) 700	417–493	703 ± 23
SC(R) 1K	670–715	1,124 ± 31
SC(R) 2K	963–1,003	1,671 ± 36
SC(R) 3K	1,190–1,216	2,671 ± 36
SC(R) 4K	1,395–1,422	3,937 ± 66
SC(R) 5K	1,606–1,638	4,991 ± 25
SC(R) 6K	1,749–1,768	6,159 ± 177
SC(R) 7K	1,808–1,824	7,382 ± 39
SC(R) 9K	2,006–2,037	8,674 ± 59
SC(R) 10K	2,187–2,228	10,002 ± 126
SC(R) 11K^∗^	2,299–2,331	11,102 ± 114
SC(R) 12K^∗^	2,392–2,417	12,007 ± 89
SC(R) 13K^∗^	2,501–2,533	13,145 ± 115


**FIGURE 2:**
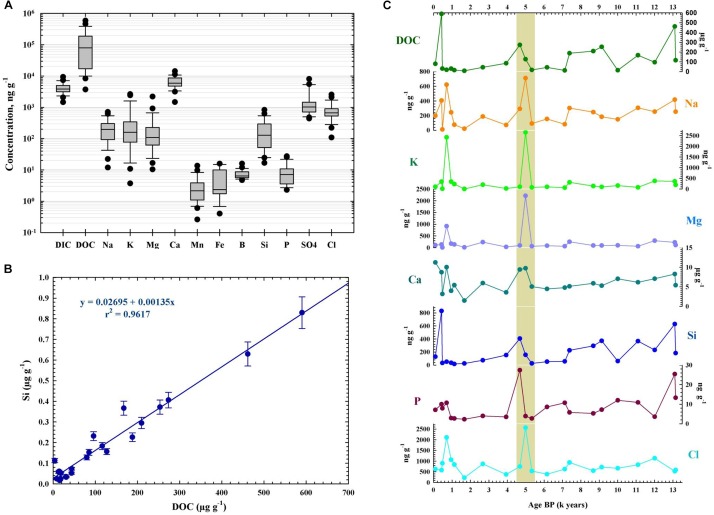
Geochemical profile of Scarisoara cave ice block. The pH, dissolved organic carbon (DOC), dissolved inorganic carbon (DIC), and elemental contents (Ca, Na, K, Fe, Mn, Mg, Si, P, B, Cl, sulfate) were measured as indicated in the Section “Materials and Methods.” **(A)** Concentration (mean ± SD) of geochemical parameters for the entire ice block. **(B)** Correlation between Si and DOC contents from Scarisoara ice core. **(C)** Distribution profile of geochemical compounds across the 13,000-year-old ice core.

*The age (average and standard deviation values) of each sample was calculated from ^*14*^C radiocarbon dating (Perşoiu et al., 2017) and analysis (Figure 2), taking into consideration the depth interval of ice core fragments. (^∗^) extrapolated values from the measured ^*14*^C radiocarbon data.*

Selected ice core samples were thawed (300 mL melted ice per sample) at 4°C and the microbial biomass was collected by filtration using 0.22 μm sterile MF-membranes (Merck Millipore, Germany) under aseptic conditions, with a vacuum-driven, stainless steel filter system (Merck Millipore, Germany). Filters were placed in 500 μL RNAlater RNA Stabilization Reagent (Qiagen, United States) and stored at -20°C until used for DNA and RNA extraction. All biomass filtering and DNA/RNA purification steps were carried out under sterile conditions using a microbiological biosafety cabinet to avoid contamination. The filtered water was stored at 4°C in 50 mL plastic conical tubes (Deltalab, Spain) for chemical analyses.

### Chemical Analyses

The pH of the ice samples was measured using a FiveEasy F20 Benchtop meter (Mettler Toledo, United States). Total carbon (TC) and Dissolved Organic Carbon (DOC) contents were measured according to US-EPA 9060A (2004), using a Formacs^HT^ Total Organic Carbon Analyzer (Skalar Analytical B.V., Netherlands), and dissolved inorganic carbon (DIC) was calculated by subtraction of DOC from TC values. For chemical measurements, samples were acidified to pH 2 using 60% Ultrapur^®^ nitric acid (Merck, Germany). Sulfate concentrations were measured by ASTM-D516-07 (ASTM 1995) standard method, using a UV/VIS double beam Lambda 25 spectrophotometer (PerkinElmer, United States). A SANGAMON-03 (Environment, Canada) certified reference was used to verify the accuracy of measurements. Concentrations of other chemicals were measured using a NexION 300S (PerkinElmer, United States) ICP-MS system equipped with a S10 Autosampler, following the US-EPA 6020B (2014) standard reference. Concentrations of calcium were estimated in dynamic reaction cell (DRC) mode using ammonia, while those of sodium, potassium, magnesium, manganese, and iron were measured in KED (Kinetic Energy Discrimination) mode, with helium as inert gas. Boron, silicon, total phosphorous and chloride concentrations were determined using the standard mode. Calibration was performed with High-Purity Standards^TM^ (HPS – Charleston, United States) NIST 1640a, NIST 1643f. The uncertainty of all analytical measurements (Supplementary Table S1) was estimated according to the ISO 11352:2012 standard.

### Flow Cytometry

The total and viable cell density of cave ice core samples were measured by flow cytometry, using a BD Accuri C6 Plus system (BD Biosciences, United States). For assessment of the total microbial community, ice samples (1 mL) were thawed at 4°C under aseptic conditions, incubated with 0.1% Tween 80 (Sigma-Aldrich, Germany) for 10 min at 37°C in a Sonorex Digital 10P ultrasonic bath (Bandelin, Germany) (2 cycles, 5% power) to disperse cellular aggregates, and labeled with 1 × SYBR Green I (SG) (Lonza Group, Switzerland). For the viable cell measurements, 100 μL of freshly thawed ice was incubated for 15 min with 1 μg mL^-1^ propidium iodide (PI) (Thermo Fisher Scientific, Germany) for quantition of dead cells (Grégori et al., 2018). The density of viable cells was calculated by subtracting the number of PI-labeled cells (dead) from the number of SG-labeled (total) ones. Unstained samples were used as a negative control for both types of labeling. Microbial cell density was expressed as number of cells per mL of melted ice. Cell viability was calculated as percentage of the measured viable cell density from the total cell content. Differences in mean cell density values were analyzed using one-way Analysis of Variance (ANOVA). Tukey’s test was performed for *post hoc* comparisons, with statistical significance of *p* > 0.05.

Theoretical metabolic rates were calculated in accordance with Price and Sowers (2004), based on measured concentrations of DOC (Supplementary Table S1), viable cell density (Table 2) values for each ice strata, the calculated ages of ice layers (Table 1) and the average carbon mass per cell value (86 fg) for terrestrial aquifers (Whitman et al., 1998). Metabolic rate estimates were expressed as grams of carbon incorporated into cell material per gram of total biomass carbon per hour. The metabolism type was assessed in comparison with corresponding metabolic rate values measured at ∼0°C (Price and Sowers, 2004).

**Table 2:** Microbial cell density from Scarisoara cave ice core.

Sample code	Total cell density (cell mL^-1^) × 10^3^	Viable cell density (cell mL^-1^) × 10^3^	Cell viability (%)
SC(R)100	3.53 ± 0.01	1.63 ± 0.45	46.2
SC(R)400	9.44 ± 3.19	5.44 ± 4.86	57.6
SC(R)700	17.55 ± 1.49	10.80 ± 2.26	61.5
SC(R)1K	14.91 ± 2.31	4.20 ± 2.56	28.2
SC(R)2K	6.69 ± 1.83	3.93 ± 1.99	58.8
SC(R)3K	11.60 ± 5.63	6.57 ± 5.91	56.7
SC(R)4K	2.02 ± 0.73	1.31 ± 0.81	64.8
SC(R)5K	0.84 ± 0.30	0.37 ± 0.34	44.7
SC(R)6K	31.44 ± 14.35	19.48 ± 14.57	62.0
SC(R)7K	3.06 ± 0.87	1.68 ± 0.99	55.1
SC(R)9K	3.77 ± 0.43	1.46 ± 0.72	38.8
SC(R)10K	1.17 ± 0.56	0.64 ± 0.60	54.7
SC(R)11K	16.48 ± 3.49	14.00 ± 3.64	84.9
SC(R)12K	24.66 ± 2.27	19.84 ± 2.45	80.5
SC(R)13K	21.00 ± 5.38	10.00 ± 5.48	47.6


*The microbial cell density of total (SC) and viable [SC(R)] communities was measured by flow cytometry, as indicated in the Section “Materials and Methods.” The mean ± SD and standard deviation values were calculated for the triplicates of the 15 ice samples. Cell viability was calculated as percentage of the viable cells from the total microbial cell density.*

### DNA and RNA Extraction and 16S rRNA Gene Illumina Sequencing

Genomic DNA (gDNA) was isolated in triplicate from distinct ice samples of the same age using the DNeasy Blood & Tissue Kit (Qiagen, United States), following the standard protocol modified with an initial lysis step. The cells (0.1–0.9 g microbial cells) were disrupted by incubation for 12 min at 20°C with innuSPEED Lysis Tube X beads (Analytik Jena, Germany) using a SpeedMill PLUS homogenizer (AnalytikJena, Germany) at 50 Hz. Lysates were then processed according to the manufacturer’s procedure.

RNA isolation was performed using the All Prep DNA/RNA kit (Qiagen, United States), following the manufacturer’s protocol modified with the preliminary mechanical lysis step described above. The RNA (12–60 ng at a concentration of 0.24–1.2 ng⋅μl^-1^) was isolated from a mixture of equal volumes (100 mL each) of the triplicate melted ice samples used for gDNA extraction. After purification, RNA samples were treated with a DNA-free DNA removal kit (Invitrogen, United States) to eliminate contaminating DNA. Reverse transcription of 6–15 ng RNA (0.75–1.87 ng⋅μl^-1^) was carried out based on the Random Hexamer amplification protocol of the Tetro cDNA Synthesis kit (Bioline, United States), using a Mastercycler proS vapo protect PCR system (Eppendorf, United States). DNA, RNA, and cDNA concentrations were measured using a Qubit fluorometer (Thermo Fisher Scientific, Germany).

16S rRNA gene and cDNA fragments (V3-V4 variable region) were PCR amplified using the Illumina prokaryotic primer pair 341F/806R (Takahashi et al., 2014) at McGill University and Génome Québec Innovation Centre, Canada. The library preparation and barcoded amplicons sequencing were carried out using the Illumina MiSeq PE300 platform (Génome Québec Innovation Centre, McGill University, Canada).

The raw 16S rRNA gene sequences of the gDNA samples and cDNA samples from Scarisoara ice core were deposited in the Sequence Read Archive (SRA) under the accession number SRP157726.

### Sequence Analyses

Raw gDNA and cDNA sequences were demultiplexed and quality filtered using QIIME 1.9.1 (Caporaso et al., 2010). Sequences were clustered into Operational Taxonomical Units (OTUs) at 99% using the Open-reference approach, based on the SortMeRNA/SumaClust algorithms (Mercier et al., 2013). Chimeras were detected using the chimera.vsearch pipeline of Mothur v.1.39.5 software (Rognes et al., 2016). Diversity indices were calculated by running the QIIME workflow Script core_diversity_analyses.py (Pylro et al., 2014). Data rarefaction was obtained using a cutoff of 23,566 sequences for gDNA, and 21,089 sequences for cDNA. Principal coordinate analysis (PCoA) with Bray–Curtis dissimilarities (Paliy and Shankar, 2016) was used for beta-diversity assessment. Permutational analysis of variance (PERMANOVA) and analysis of similarities (ANOSIM) tests were carried out using *MicrobiomeAnalyst* platform (Dhariwal et al., 2017) to determine statistical differences between *a priori* groups. Linear discriminant analysis effect size (LEfSe) (Segata et al., 2011) was used to explain the differences between the total and potentially active bacterial communities. Significant features were considered for an adjusted *p*-value < 0.05. Microbial community profiles of the 16S rRNA gene sequences and graphical visualization were conducted using *Microbiome Analyst* (Dhariwal et al., 2017) with the default parameters for counter filters (4), prevalence in samples (20%), and inter-quartile variance filter. Data were normalized using the Total Sum Scaling (TSS) method (Dhariwal et al., 2017). Heatmaps were generated in R using the Phyloseq package (McMurdie and Holmes, 2013). Assigned cDNA OTUs not found in the corresponding gDNA library (Supplementary Table S2) represented 0.0009–0.912% of the total OTUs (average of 0.23%) and were indicative of a reduced putative contamination during RNA processing. In order to reduce bias, the OTUs present and only assigned from cDNA libraries were eliminated from sequence analyses.

## Results

### Geochemical Profile of Scarisoara Ice Core

Geochemical analyses of the sequential ice core samples revealed a heterogeneous pH distribution and variable concentrations of organic and inorganic carbon and chemical components across the cave ice block (Supplementary Table S1). The ice core had a slightly alkaline pH in the 6.91–9.59 interval (with a mean pH value of 8.45 ± 0.42). The mean values of dissolved organic carbon (DOC) and dissolved inorganic carbon (DIC), and of the chemical concentration along the ice core (Figure 2A) were similar to those found in other ice caves (Clausen et al., 2006; Kern et al., 2011). Concentrations of the total dissolved solids (Supplementary Table S1) varied between 2.28 and 21.71 μgg^-1^, with an average of 9.84 ± 4.91 μgg^-1^. The main component was calcium, as expected for a limestone ice cave, with values ranging from 1.50 to 11.23 μgg^-1^ (average 6.24 ± 2.39 μgg^-1^). Sodium, potassium, magnesium, sulfates, and chlorides were the major constituents throughout the ice core, while manganese, iron, boron and phosphorus were present in lower concentrations (Figure 2A). Silicon, usually a minor component of environmental waters, was present in high concentrations in all ice layers, exhibiting similar concentrations to those of sodium, potassium, magnesium and chloride (Figure 2A). An average DOC concentration of 130.89 ± 14.88 μgg^-1^ was calculated for the 25-m cave ice core, with considerable variations (8.32–590.08 μgg^-1^) between strata (Supplementary Table S1). Silicon and DOC content across the cave ice core were tightly correlated (Figure 2B), indicating a prevailing external origin of the organic carbon input. The chemistry profile of the ice block (Figure 2C) showed a non-homogenous temporal distribution. Higher values occurred in ice layers deposited during the last millennium, in addition to a spike in all the chemical components during the 4,500–5,000 cal BP period. Slightly increased concentrations of DOC, Si, Ca, P, and Na were also observed in ice strata formed 7,000 years ago and in older ice deposits (Figure 2C).

### Microbial Cell Density Across the Cave Ice Block

The microbial cell density across the cave ice core as determined by flow cytometry indicated high levels of variation between ice strata, with average values ranging from 0.84 × 10^3^ cells⋅mL^-1^ to 3.14 × 10^4^ cells⋅mL^-1^ (Table 2). The microbial cell density of the cave ice core was dissimilar (Supplementary Table S3), showing statistically significant differences [*F*_(14,30)_ = 13.49, *p* < 0.0001] between strata. The highest cell abundance was found in the 6,000-year-old ice (sample SC6K, 3.14 × 10^4^ cells⋅mL^-1^), followed by ice formed during 11,000–13,000 cal BP (samples SC11K, SC12K, and SC13K of 1.64 × 10^4^ cells⋅mL^-1^, 2.47 × 10^4^ cells⋅mL^-1^ and 2.1 × 10^4^ cells⋅mL^-1^, respectively) and 700–1,000 cal BP of 1.75 × 10^4^ cells⋅mL^-1^ (SC700) and 1.49 × 10^4^ cells⋅mL^-1^ (SC1K). The lowest cell density (0.84 × 10^3^ cells⋅mL^-1^) occurred in the 5,000-year-old ice (SC5K), closely followed by 100, 4,000, and 10,000-year-old ice containing 1.17 × 10^3^ cells⋅mL^-1^ (SC10K), 2.02 × 10^3^ cells⋅mL^-1^ (SC4K), and 3.53 × 10^3^ cells⋅mL^-1^ (SC100). The cell density of the viable community (Table 2) ranged between 3.7 × 10^2^ cells⋅mL^-1^ and 1.98 × 10^4^ cells⋅mL^-1^ (Table 2), with significant differences [*F*_(14,30)_ = 5.78, *p* < 0.0001] between strata (Supplementary Table S3). Higher values (in the 10^4^ cells⋅mL^-1^ range) were found in SCR700, SCR6K, SCR11K, SCR12K, and SCR13K samples, and the lowest abundance (in the 10^2^ cells⋅mL^-1^ range) in SCR5K and SCR10K. The corresponding cell viability (Table 2) across the ice core showed large variations from 28.2% (SCR1K) to 84.9% (SCR11K) in the potentially active microbial community. Both total and viable communities showed a slight overall increase with age of the perennial ice deposits (Supplementary Figure S2A). Meanwhile, microbial abundance varied considerably across the ice core (Supplementary Table S3), with a decline from 700 to 5,000 cal BP, a spike at 6,000 cal BP, and a prominent increase after 10,000 cal BP for the total and the viable communities. A slight increase in microbial cell viability (0.0011 cells⋅mL^-1^ 10^3^ year^-1^) was recorded in the cave ice deposits from the last 13,000 years (Supplementary Figure S2B).

### Diversity of Total and Potentially Active Bacterial Communities From the Cave Ice Block

The community composition and diversity of total (gDNA) and putative active (cDNA) bacterial communities from the ice core selected samples was assessed by 16S rRNA gene amplicon Illumina sequencing. For the gDNA library, a total of 6,037,525 post quality control filtered reads were obtained, with 402,501 mean reads per sample (Table 3). The number of observed OTUs ranged between 496 and 4,047, with a median of 2,546 OTUs. High Shannon index values (7.22–7.83) were found for ice layers accumulated during the last two millennia (samples SC100, SC400, SC700, SC1K, and SC2K), while a much a lower alpha diversity (Shannon index 3.15–3.76) characterized the SC4K, SC6K, and SC11K prokaryotic communities (Table 3). The corresponding Chao1, Fisher and Inverse Simpson indices supported this diversity profile that showed no major variations with the age of ice across the ice core (Table 3).

**Table 3:** Number of reads, OTUs and diversity indices for total (gDNA), and potentially active (cDNA) bacterial communities.

	Sample	Subsampled reads	OTUs	Alpha diversity index			
				Shannon	Chao1	Fisher	Inv simpson
gDNA	SC100	149,993	2,227 (923–3,694)	7.83 (7.07–8.57)	2,842 (1,292–4,555)	376 (144–645)	91.01 (72.35-105.13)
	SC400	194,124	2,822 (2,266–3,223)	7.22 (7.09–7.30)	4,399 (3,627–5,065)	472 (376–532)	47.01 (45.39-48.16)
	SC700	217,426	4,047 (3,519–4,442)	7.51 (6.92–7.88)	5,786 (5,218–6,350)	713 (605–780)	44.21 (20.88-63.88)
	SC1K	112,974	2,830 (2,109–3,867)	7.43 (7.34–7.49)	3,977 (3,041–5,117)	534 (376–762)	34.80 (26.31-42.98)
	SC2K	77,590	1,998 (1,526–2,773)	7.43 (6.86–7.97)	2,560 (2,096–3,359)	389 (255–581)	26.24 (18.40-32.41)
	SC3K	117,210	1,987 (731–3646)	5.41 (4.50–6.66)	2,987 (1,450–4,887)	359 (108–714)	12.80 (9.54-18.01)
	SC4K	145,156	1,390 (724–2,468)	3.46 (1.76–5.78)	1,938 (1,125–3,140)	219 (102–410)	6.06 (1.43-14.69)
	SC5K	107,304	2,974 (2,656–3,135)	6.56 (6.42–6.65)	4,449 (4,108–4,632)	573 (532–600)	19.67 (18.53-20.83)
	SC6K	44,773	496 (359—-691)	3.15 (2.77–3.48)	887 (719–793)	80 (51–114)	2.94 (2.67-3.21)
	SC7K	132,563	1,938 (1,552–2,545)	6.1 (5.79–6.55)	2,924 (2,576–3560)	327 (249–453)	28.09 (24.85-32.55)
	SC9K	185,112	2,714 (2,148–3,188)	5.66 (5.36–5.92)	3,872 (3,220–4,397)	455 (355–544)	14.36 (13.12-15.56)
	SC10K	78,359	1,364 (473–2,555)	5.18 (3.90–7.19)	1,996 (999–3,238)	251 (69–512)	22.67 (6.15-54.12)
	SC11K	90,282	1,009 (472–1963)	3.76 (1.18–5.52)	1,408 (749–2,402)	163 (69–328)	6.64 (1.39-10.56)
	SC12K	166,300	2,716 (2,497–3,138)	6.15 (5.95–6.39)	4,394 (4,054–5,033)	469 (413–578)	24.35 (22.59-26.03)
	SC13K	193,340	3,424 (2,349–4,182)	6.51 (4.98–7.36)	4,701 (3,473–5,468)	602 (385–762)	25.28 (8.74-33.97)

cDNA	SCR100	77,663	462	3.27	881	66	4.33
	SCR400	119,430	2,008	5.72	2863	346	12.21
	SCR700	78,801	444	3.14	998	63	4.37
	SCR1K	22,560	261	3.41	606	42	4.88
	SCR2K	23,363	415	4.19	699	73	5.50
	SCR3K	44,379	393	3.49	956	60	4.83
	SCR4K	24,510	585	4.27	887	109	6.21
	SCR5K	73,092	2,426	6.93	3346	488	17.79
	SCR6K	115,586	642	3.53	1113	90	4.95
	SCR7K	139,685	1,946	5.36	2691	323	11.04
	SCR9K	183,793	2,895	6.27	3798	493	20.43
	SCR10K	170,252	883	3.80	1423	123	5.62
	SCR11K	132,818	2,056	5.62	2510	348	12.41
	SCR12K	26,326	470	4.37	753	82	6.45
	SCR13K	57,949	243	7.62	3451	520	35.08


*Subsampled reads, OTUs and alpha diversity indices (average, minimum, and maximum) are indicated for triplicate gDNA and cDNA samples.*

Analysis of the bacterial cDNA sequences were able to recover 1,290,207 post quality control filtered reads, with 86,013 mean reads per sample (Table 3). The number of OTUs varied between 243 in the oldest analyzed ice (SCR13K) and 2,895 in the 9,000-year-old ice (SCR9K), with a median of 585 OTUs. Shannon index values ranged between 3.14 (SCR700) and 7.62 (SCR13K) (Table 3). Similar to the gDNA samples, the variability of the Shannon, Chao1, Fisher, Inverse Simpson indices for cDNA libraries, in accordance with that of the number of observed OTUs, revealed an unequal distribution of viable bacterial diversity along the ice core (Table 3). Moreover, Shannon, Chao1, Fisher, and Inverse Simpson diversity indices calculated for gDNA and cDNA samples showed significant differences (Supplementary Figure S3), in support of a reduced diversity of the putative active bacterial community across the ice block.

**FIGURE 3:**
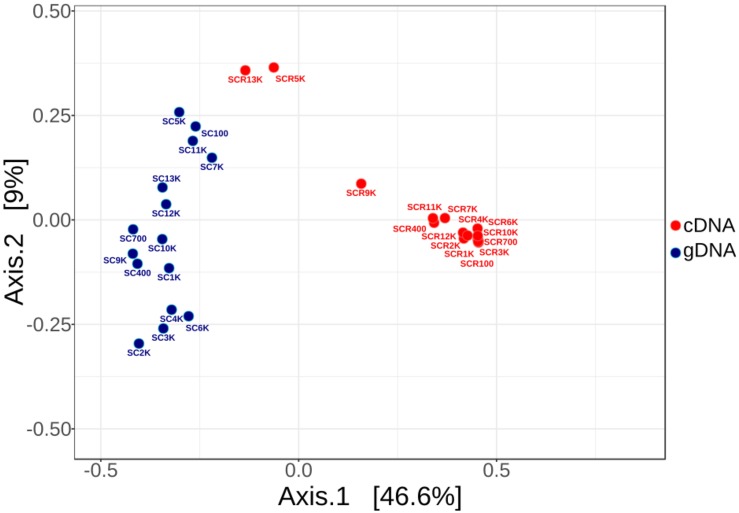
Principal component ordination analysis (PCoA) of the prokaryotic OTUs distribution across the 13,000-year-old cave ice core. Variation of the 16S rRNA amplicons from gDNA (triplicates of 15 SC samples) and cDNA (15 SCR samples) Illumina data was analyzed for the total (red) and potentially active (blue) communities, using average OTU values of the gDNA triplicate libraries. (Pseudo-F: 21.46; *R*^2^: 0.43; *p* < 0.001 by PERMANOVA).

Rarefaction curves of both gDNA and cDNA libraries (Supplementary Figure S4) indicated a partially identified microbial community for both gDNA and cDNA libraries. Principal component ordination analysis (PCoA) of total (SC) and potentially active (SCR) bacterial OTUs distribution across the ice block (Figure 3) explained 55.6% of their variance. The clearly separate clusters of the gDNA and cDNA OTU libraries indicated a difference in composition of the total and potentially active communities. No age-dependent distribution of the microbial community for both gDNA and cDNA libraries was observed along the ice core. Interestingly, the potentially active bacterial communities from 5,000 (SCR5K) and 13,000 (SCR13K) year-old ice formed a discrete group, suggesting a distinct microbial structure of the viable microbiome in these ice strata. Also, no significant differences (PERMANOVA F: 15.296; *R*^2^: 0.88437; *p* < 0.25) were observed between the total (SC5K) and the potentially active (SCR5K) bacterial communities contained in the 5,000-year-old cave ice (Figure 3).

### Taxonomic Patterns and Temporal Distribution

Bacterial OTUs constituted 99.18% of the total prokaryotic community estimated across all sections of the core, less than 0.45‰ were archaeal OTUs and 0.82% corresponded to unassigned phylotypes. A total of 35 bacterial phyla, 88 classes, and 526 genera were identified in the total community (gDNA). The potentially active bacterial community (cDNA) was composed of 25 phyla, 58 classes, and 325 genera. In addition, limited archaeal taxa (2 phyla, 2 classes, and 2 genera) were detected in both the communities combined.

**FIGURE 4:**
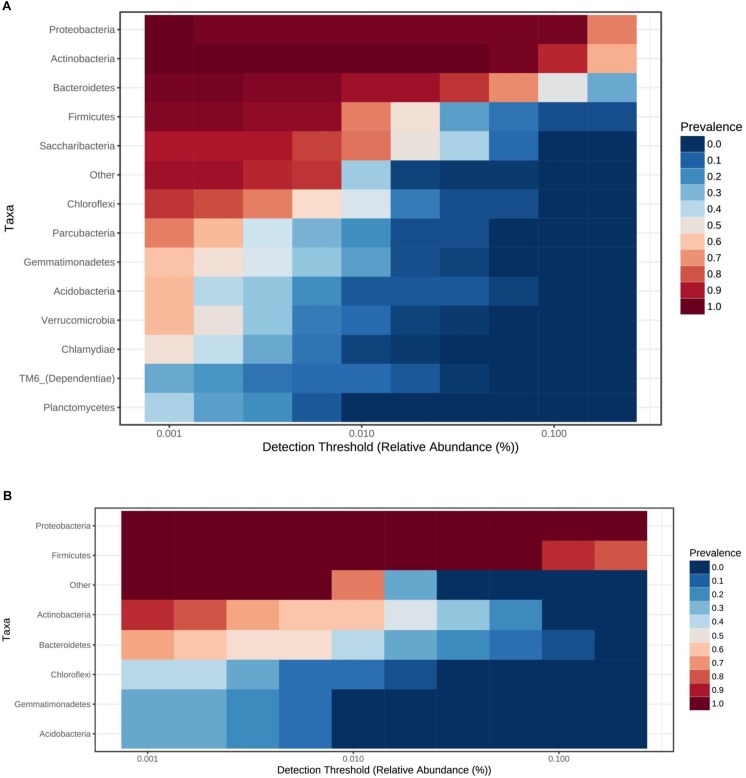
Ice core bacterial community composition at phyla level. Prevalence of bacterial phyla from Scarisoara ice block of the **(A)** total bacterial community (gDNA), and **(B)** potentially active bacterial community (cDNA).

Total bacterial phyla from the cave ice block (Figure 4A) revealed the dominance of Actinobacteria (38.5%), and Proteobacteria (33.5%), followed by Bacteroidetes (12.9%), and Firmicutes (6.2%). Saccharibacteria (2.7%) and Chloroflexi (1.4%) were only scarcely represented. The incidence of Parcubacteria, Gemmatimonadetes, Acidobacteria, Verrucomicrobia, Chlamydiae, TM6, and Planctomycetes phyla was also extremely low (0.2–1%). The identified cDNA prokaryotic community was largely represented by Proteobacteria (52.5%), and Firmicutes (38.5%) (Figure 4B). Unlike the total bacterial community, Actinobacteria and Bacteroidetes occupied only 3.1 and 2.6% of the potentially active fraction. The relative abundance of Chloroflexi, Gemmatimonadetes and Acidobacteria phylotypes was >0.2% across the cave ice block. Unassigned taxa represented 0.8% of the total bacterial community and 1.4% of the putative active fraction. The prevalent bacterial OTUs from the gDNA library belonged to *Cryobacterium, Lysinomonas, Pedobacter*, and *Aeromicrobium* genera (Supplementary Figure S5). Meanwhile, the potentially active (cDNA) community was dominated by *Clostridium sensu stricto, Pseudomonas, Janthinobacterium, Stenotrophomonas*, and *Massilia* genera, revealing a composition distinct from that of the gDNA (Supplementary Figure S5).

**FIGURE 5:**
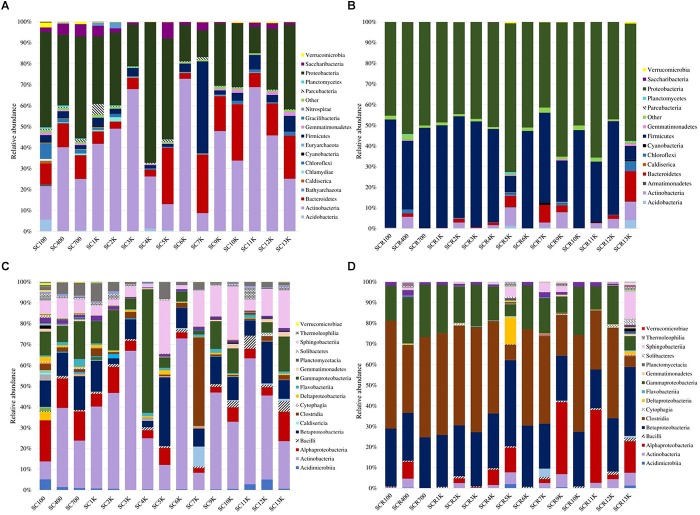
Bacterial taxa distribution across the 13,000-year-old cave ice chronosequence. Relative abundance of bacterial phyla from **(A)** total (gDNA) and **(B)** potentially active (cDNA) communities; bacterial community structure at class level from **(C)** total (gDNA) and **(D)** potentially active (cDNA) communities.

The microbial chronosequence revealed a dissimilar taxa profile along the cave ice core. A divergent distribution of bacterial phyla from the total (gDNA) microbial community was observed in all ice strata (Figure 5A,C). Meanwhile, the putative active (cDNA) community showed a more uniform distribution in all strata (Figure 5B,D). Total (gDNA) bacterial taxa (Figure 5A) were dominated by Proteobacteria and Actinobacteria in most ice layers, except for SC7K, which was largely represented by Firmicutes (43.8%). The relative abundance of Proteobacteria showed a decreasing trend over the last 7 millennia from 48.7 to 12.7%, followed by a slight increase up to 40.3% in ice strata older than 7,000 years (Figure 5A). Samples SC4K and SC5K showed a substantial change in bacterial community composition, with a strong increase to 66.9% of Proteobacteria. Meanwhile, Actinobacteria relative abundance showed an increasing trend during the last 6,000 years up to 72.8% (SC6K), followed by a decline up to 24.8% in older strata. A dramatically reduced relative content of Actinobacteria taxa was observed in SC4K (24.9%) and SC5K (12.4%). Bacteroidetes were mainly present (20.5–27.9%) in SC5K, SC7K, SC10K, and SC13K ice, but only constituted 2.7% in SC6K (Figure 5A). The potentially active (cDNA) bacterial community was mostly divided between Proteobacteria (41.2–65.6%) and Firmicutes (20.1–52.5%) but with a more homogenous distribution along the ice core (Figure 5B). However, the viable community from SCR5K was dominated by Proteobacteria (71.9%), and SCR13K showed a relatively high Bacteroidetes content (14.6%). Moreover, Firmicutes relative abundance was reduced to 6.8% (SCR13K) and 7.9% (SCR5K).

At the level of class (Figure 5C), Actinobacteria dominated the total (gDNA) bacterial communities from most ice strata (22.7–72.4%), with the highest relative abundance recorded in SC6K, and lower values recorded from SC100 (8.5%), SC5K (11.7%), and SC7K (8.2%). Acidimicrobiia was mainly present (2.7–5.1%) in either recently formed ice SC100 (5%) or old ice layers SC11K (2.8%) and SC12K (5.1%). Bacteroidetes was represented by class Sphingobacteriia throughout the entire ice core. Meanwhile, Bacteroidia were mostly found in SC7K (9.6%) and Cytophagia in SC12K (1.4%) ice layers. Clostridia (Firmicutes) was the dominant class in SC7K (42.3%). Betaproteobacteria OTUs were present in all ice strata, dominating the SC5K (33%) ice sample, but were scarcely present in SC2K (2.6%) and SC4K (3.02%). Gammaproteobacteria prevailed in SC4K (59.2%), and Alphaproteobacteria had a high representation (6.1–19.4%) in ice deposits formed during the last 2,000 years and in the oldest SC13K (14.2%) ice layer (Figure 5C). In the potentially active community (Figure 5D), Clostridia (Firmicutes) was the major bacterial class in all layers, except for SCR5K (7.3%) and SCR13K (5.1%), with the highest relative abundance seen in SCR100 (52.2%). Most of the ice core strata were characterized by a high Gammaproteobacteria (5.6–24.5%) and Betaproteobacteria (18.7–41.5%) relative content. Meanwhile, Deltaproteobacteria had an important input (12.9%) in SC5K, and Alphaproteobacteria taxa were dominant (35%) in SCR9K and SCR11K.

**FIGURE 6:**
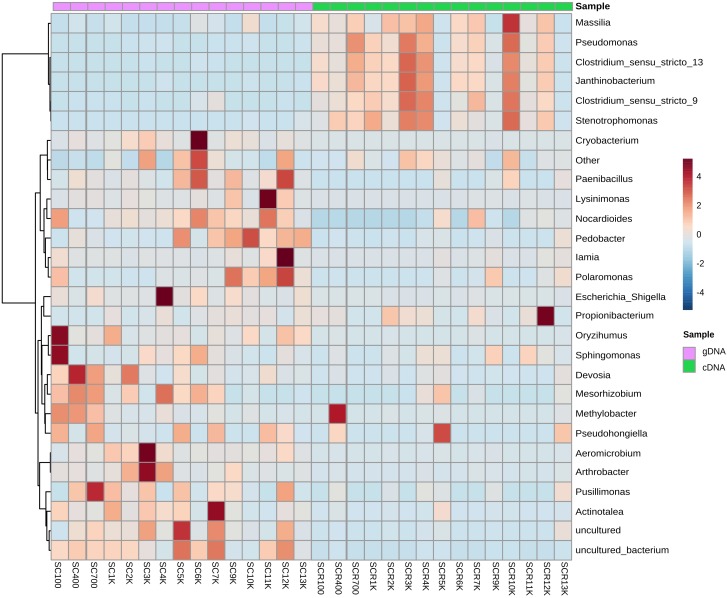
Heatmap analysis of the cave ice prokaryotic community composition at genera level from the (purple) gDNA (total community) and (green) cDNA (potentially active community) replicate samples.

Bacterial genera were heterogeneously distributed throughout the ice block strata, with distinct prominent taxa in the gDNA and cDNA community libraries (Figure 6). Among the dominant bacterial genera from the total community (Supplementary Figure S5), *Cryobacterium* was mostly present in SC6K (61.5%)*, Lysinimonas* in SC11K (52.2%), and *Pedobacter* in older (>7,000 cal BP) ice strata (4.03–24.4%). Both *Aeromicrobium* and *Arthrobacter* genera prevailed in SC3K, representing 23.7 and 3.67%, respectively. Moreover, *Paenibacillus* OTUs had the highest occurrence in SC6K (0.87%) and SC12K (0.79%). The 4,000-year-old ice layer (SC4K) was rich in *Escherichia* and *Shigella* OTUs (56.1%). The actinobacterial *Iamia* (2.6%) and *Actinotalea* (1.2%) phylotypes were mainly present in the 12,000 and 7000-year-old ice layers, respectively. Among those genera generally recorded from more recently formed ice strata*, Sphingomonas* and *Oryzihumus* were dominant in SC100 (9.65 and 2.98%, respectively), *Devosia* in SC400 (5.9%), SC700 (3.5%), and SC2K (2.3%), and *Pusillimonas* in the SC700 ice layers (7.5%). A high relative abundance of unidentified species was found in SC5K, SC7K, and SC12K. The dominant genera within the identified cDNA library (Supplementary Figure S5) were more uniformly distributed across the cave ice block (Figure 6). SCR10K bacterial community was dominated by *Pseudomonas* (21.1%), *Clostridium sensu stricto 9* (4.6%), *Clostridium sensu stricto 13* (41.4%), *Janthinobacterium* (13.9%), and *Stenotrophomonas* (1.9%) genera. These taxa also inhabited ice deposits formed during the last four millennia, in particular the SCR3K and SCR4K samples. *Massilia* (Proteobacteria) species were mostly found in SCR12K (0.89%). *Methylobacter, Pseudohongiella*, and *Propionibacterium* species were populating the 400, 5,000 and 12,000-year-old ice viable communities (Figure 6).

The rare (<1‰) archaeal taxa identified in Scarisoara cave ice core (Supplementary Table S4) belonged to phyla Bathyarchaeota (SC100) and Euryarchaeota (SC100, SC400, SC5K, SC7K, SC9K, and SC12K) represented by classes Methanomicrobia and Thermoplasmata. Among potentially active archaeal taxa, Euryarchaeota phylotypes of *Methanosphaerula* and *Methanosarcina* (Methanomicrobia) were present in 2,000 (SCR2K) and 5,000 (SCR5K) year-old samples.

## Discussion

### Unique Bacterial Variability in Scarisoara Cave Ice Chronosequence

This characterization of total and potentially active bacterial communities from a 13,000-year-old perennial ice core of Scarisoara Ice Cave revealed a highly diverse microbiome in cave ice strata formed since the Late Glacial period, representing a pioneering study for this type of habitat. A comparable bacterial diversity (median Shannon index of 5.9) of the 400 and 900-year-old ice from the edge of the ice block in Scarisoara Ice Cave (Itcus et al., 2018) and the ice core samples of the same age collected from the central part of the ice block (this work) was observed, indicating a homogenous spatial distribution of the ice microbial community. A heterogeneous chronological distribution of prokaryotic communities was detected across the cave ice block. This corresponded to a 2.5-fold Shannon index variation along the 13,000-year-old ice core, from the lowest diversity in 6,000 cal BP ice strata to higher values in older ice strata. Similar microbial diversity (average Shannon index 5.92) was also found in Jinjia limestone cave sediments from Loess Plateau, China (Wu et al., 2015). Meanwhile, a significantly higher diversity (average Shannon index 8.2) was reported in glacier ice from Alaska Lemon Creek Glacier (Sheik et al., 2015).

A complex potentially active prokaryotic community that preserved 80% of the total microbial community alpha diversity appeared to populate the underground Scarisoara glacier. In comparison with other caves and frozen habitats, Scarisoara perennial ice contained a unique prokaryotic community characterized by a complementary distribution of Actinobacteria and Proteobacteria taxa in most strata. The presence of Bacteroidetes and Firmicutes phyla was also featured in the total (gDNA) communities, with a higher incidence in older ice layers. Actinobacteria, one of the main taxa observed in cryospheric environment microbiomes (Anesio and Laybourn-Parry, 2012), was also dominant in the 900-year-old ice strata collected from the Little Reservation area of the cave (Itcus et al., 2018), indicating a homogenous spatial distribution across the Scarisoara ice block. Ice microbiomes from the GLDD and NJKS glaciers of the Tibetan Plateau were also dominated by Actinobacteria taxa (50%), but had a twofold higher (25%) relative content of Bacteroidetes (Liu et al., 2015) than that seen in the current study. Proteobacteria OTUs from both the total and potentially active cave ice communities belonged to classes Gamma-, Beta-, and Alphaproteobacteria, similar to the rock-wall deposits and aquatic sediments collected from a Loess Plateau limestone cave in China (Wu et al., 2015).

Recent studies have shown an elevated proportion of Actinobacteria (86%) in sediments from Hawaiian lava tubes (Teehera et al., 2017), similar to that seen in the 6,000-year-old ice layer of Scarisoara cave ice. Moreover, the microbial community associated with ice deposits from this volcanic cave largely contained Proteobacteria phylotypes (39%), with no Firmicutes taxa present (Teehera et al., 2017), unlike the potentially active bacterial fraction seen in the current study. The low incidence of Actinobacteria in 4,000–5,000-year-old strata from Scarisoara Ice Cave were associated with high contents of organic carbon and salts, which may have resulted in an enhanced representation of Proteobacteria, in particular within the potentially active ice microbial community. A different trend was observed in Antarctic fumarolic ice cave sediments, which were characterized by an important contribution from oligotrophic microorganisms and phototrophic (Cyanobacteria and Chloroflexi) taxa (Tebo et al., 2015).

Interestingly, the putative active bacterial community from Scarisoara was characterized by a major shift between the Actinobacteria and Firmicutes phyla, and the prevalence of Beta- and Gammaproteobacteria and Clostridia phylotypes along the ice core. The dominance of potentially active *Clostridium sensu stricto 9* and *13* (Firmicutes) species, known from soils and as inhabitants of animal intestinal tracts (Maczulak, 2011) could be related to their endospore-forming capacity, as an adaptive strategy for surviving in extreme environments (Filippidou et al., 2016). Various psychrophilic and psychrotrophic bacterial species characteristic of cold environments were highly abundant in the putative active bacterial community of the cave ice block (Supplementary Table S5 and Figure 6). These species included *Massilia* sp. also identified from the Muztagh Glacier, China (Shen et al., 2015; Guo et al., 2016), and *Janthinobacterium* sp. reported from Qinghai-Tibet Plateau permafrost (Zhang et al., 2007) and tundra soil [*J. lividum* (MH929893)]. Among the ubiquitous *Pseudomonas* species, very few are known to thrive in cold environments. The presence of a putative active *P. psychrophila* in Scarisoara extended the distribution of this cold-adapted species previously found in Arctic fjord water (Abraham and Thomas, 2015) to alpine ice caves. Moreover, several sequences similar to *P. fragi, P. putida*, and *P. fluorescence* from soil and insect/cattle carriers were among the highly abundant bacteria reported in this study (Supplementary Table S5). The ice layer formed at 5,000 cal BP showed a major shift in microbial composition, and was dominated by potentially active *Pseudohongiella* sp. able to reduce nitrate (Xu et al., 2016). *Stenotrophomonas* sp. was a major constituent of the viable microbiome across the cave ice block; it also belongs to a ubiquitous genus capable of anaerobic growth based on nitrate reduction that plays an important role in the elemental cycle in nature (Heylen et al., 2007).

The abundance of Deltaproteobacteria in the potentially active community from the 5,000-year-old ice deposits revealed a possible climatic-driven switch to a more complex functional diversity of the cave ice microbiome (Claussen et al., 1999). Variation in the microbial communities during that period were associated with changes in the ice geochemical composition. These taxa favored by this physiochemical shift included anaerobic *Desulfobulbus* (sulfate-reducing bacteria), *Desulfuromonas* (sulfur-reducing bacteria), and *Geobacter* (ferric iron-reducing) phylotypes, and aerobic *Myxococcus* species capable of releasing myxospores under extreme environmental conditions.

A wide range of psychrotrophic and psychrophilic bacterial homologs were found throughout the Scarisoara cave ice block. *Cryobacterium* sp. are found in most frozen habitats (Inoue and Komagata, 1976; Liu et al., 2018) and were common in the 6,000-year-old cave ice. *Arthrobacter* and *Aeromicrobium* OTUs, commonly detected in ice caves (Margesin et al., 2004), glacier ice cores (An et al., 2010) and alpine (Zhang et al., 2010) and Antarctic (Ganzert et al., 2011) soils, dominated the Scarisoara ice layer deposited at 3,000 cal BP. Moreover, *Carnobacterium maltaromaticum* -previously reported from a 25,000-year-old permafrost ice wedge (Katayama et al., 2007) was identified in the potentially active community from our cave ice deposits (Supplementary Table S5). This soil bacterium is capable of surviving at negative temperatures for long periods and is highly resistant to freeze–thaw cycles (Walker et al., 2006), suggesting a broader distribution of such cold-adapted microorganisms in various ice-cold environments worldwide.

Phototrophic OTUs belonging to the phyla Cyanobacteria and Chlorobi were identified in the total and potentially active bacterial communities from several ice strata. Cave ice deposits older than 7,000 years contained putative active *Chroococcidiopsis* sp., one of the most primitive cyanobacteria capable of surviving under extreme environmental conditions (Baqué et al., 2013). *Gloeocapsa* sp., an ancient line of photosynthesizing bacteria involved in calcium carbonate mineralization and playing an important role in calcification processes in palaeoenvironments (Bundeleva et al., 2014), was also found in the viable community of the oldest ice strata (13,000 cal BP) from Scarisoara cave. Moreover, Chlorobi phylotypes belonging to Cytophagales/green sulfur bacterium OPB56 (Canfield et al., 2005) and uncultured SJA-28 (Hiras et al., 2015) families were identified in Scarisoara ice layers accumulated during the last millennium (SC100, SC400, SC1K samples). These taxa were previously reported from both 400 and 900-year-old ice strata collected from the Little Reservation area of this cave (Itcus et al., 2018). Chlorobium species, known as strict anaerobes tolerant of extreme temperatures (Pfennig and Overmann, 2015), were recorded in SC100, a recent stratum. The presence of potentially active Chlorobi in the 4,000–5,000 and 13,000-year-old strata characterized by high organic carbon content suggested the contribution of a phototrophic microbial community to the cave ice microbiome by performing anoxygenic photosynthesis in under extremely reduced light intensity (Raven et al., 2000). Furthermore, the presence of *Brevundimonas* sp. (Alphaproteobacteria) in both the total and potentially active communities of these strata revealed the possible existence of aerobic anoxygenic phototrophic metabolism in perennial cave ice. Thus, the potentially active microbial community from ice strata formed about five millennia ago including sulfate-reducing bacteria, aerobic and anaerobic anoxygenic phototrophs and methanogenic archaea, might correspond to a microbial mat-like structure that developed in the cave perennial ice block. The formation of such a stable microbial structure at the interface between water and ice sediments in liquid-water veins of ice deposits (Campen et al., 2003) could be associated with the high salt concentrations of the 5,000-year-old ice layer. Further investigations of this hypothesis could lead to expanding the occurrence and biogeography of microbial mats or complex photosynthetic biofilms in cold environments.

### Geochemical Impact on the Cave Ice Microbiome

The chemical composition of Scarisoara perennial ice was broadly comparable to that observed in accumulated ice deposits from other ice caves (Clausen et al., 2006; Kern et al., 2011) – however, considerable physiochemical differences were apparent relative to other glacial habitats. The cave ice concentrations were two orders of magnitude higher than those reported from the Greenland GISP2 ice core (Mayewski et al., 1997) of meteoric water origin and more than two orders of magnitude lower than those from Western Yakutia (Siberia) ground ice in close contact with the bedrock (Alexeev et al., 2016). The concentration of salts in Scarisoara ice strata likely reflected the source of accumulated perennial cave ice. The cave water originated from both advection by infiltration and dripping, and from atmospheric direct deposition through the large entrance shaft of the cave. Here, silicon is potentially sourced either from the clays in the fissure above the cave or from dust gravitationally deposited on the surface of the ice and snow (Figure 1), while organic carbon is primarily sourced from the soil above the cave (Feurdean et al., 2011). The strong correlation between Si and DOC contents along the ice core suggested the main soil origin of organic matter in the cave ice deposits is via water infiltration from the surface, which flushes the Si from rock fissures before reaching the cave. The high TDS, Si and DOC contents of the ice deposits accumulated during the last 500 years could reflect the influence of a cold climate period with frequent summer storms leading to infiltrations of large volumes of water in the cave (Perşoiu and Perşoiu, 2018). This hypothesis was sustained by the high content of vegetal macrofossils inform the cave ice deposits formed during this interval (Feurdean et al., 2011). In the 4,500–5,000-year-old ice strata, the major physiochemical shift could be related to the increased autumn-time precipitation regime advecting a higher amount of Mediterranean moisture to the cave’s region (Perşoiu et al., 2017). During that period, a major change occurred in the rate of ice accumulation in Scarisoara cave combining prolonged melting with rapid increase of ice strata. The higher precipitation regime could result in enhanced infiltrations of drip water carrying large amounts of soil-associated silicon and organic carbon, thus accounting for the high concentrations of TDS, Si, and DOC in the cave ice deposits accumulated five millennia ago.

**FIGURE 7:**
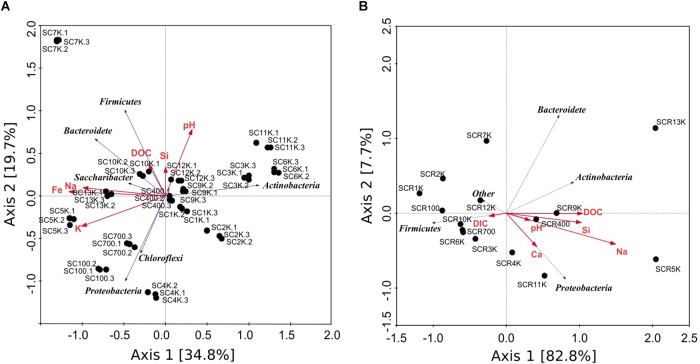
Redundancy analysis (RDA) triplot summarizing variations in the geochemistry and bacterial community across ice sample gradients from Scarisoara cave. The phyla distribution from **(A)** gDNA samples triplicates and **(B)** cDNA samples were analyzed in relation to relevant geochemical parameters pH, DIC, DOC, Ca, Na, K, Fe, Si.

Ice geochemistry appeared to influence the composition of the ice microbiome and of the potentially active bacterial community we identified in Scarisoara Ice Cave. Redundancy analysis (RDA) of phyla distribution from the total and putative active communities in relation with the geochemical parameters of ice core samples explained 88.7 and 90.5% of the variance for the gDNA and cDNA libraries, respectively (Figure 7). In both cases, the RDA canonical axes were composed of several covarying geochemical parameters including pH, DOC, and DIC contents, and Ca, Na, K, Cl, Si, and Fe concentrations, with dissimilar correlation between phyla distribution of the total (Figure 7A) and potentially active (Figure 7B) prokaryotic communities with the ice chemistry, in particular for Firmicutes, Proteobacteria and Actinobacteria,. Changes in phyla distribution correlations with the DOC/DIC contents were observed between the total (gDNA) community (Figure 7A) and the potentially active (cDNA) microbiome (Figure 7B), suggesting that carbon type (organic and inorganic) was among the drivers of their microbial composition. Thus, potentially active Firmicutes phylotypes showed a negative correlation with DOC, while being positively correlated with DIC concentrations, conversely to the pattern observed for Proteobacteria and Actinobacteria OTUs distribution. Since many representatives of Firmicutes, Actinobacteria and Proteobacteria include nitrogen fixing organisms (Boyd and Peters, 2013; Nash et al., 2018) and some Firmicutes and Proteobacteria are capable of CO_2_ fixing (Tabita et al., 2007), the correlation of the DIC contents with the Firmicutes distribution in the cave ice block suggested that *Clostridium* sp., one of the major taxa in the potentially active community (Supplementary Figure S5), could play a key role in the food web of cold environments characterized by low organic carbon content (Tveit et al., 2012). The cellulose-fermenting processes carried out by such species produce CO_2_, H_2_, organic acids and ethanol, which could potentially serve as growth substrates for other bacteria (Leschine and Canale-Parola, 1989; Alam et al., 2006).

The Scarisoara cave ice block harbored a relatively low abundance (10^3^ cells⋅mL^-1^–10^4^ cells⋅mL^-1^) microbial community that varied across the 13,000-year-old strata. Both microbial cell density and the viability of the total and potentially active communities showed a slight increase with the age of the ice layers. However, microbial abundance in the central part of the ice block located in the Great Hall (Figure 1) appeared to be about 40-fold lower than that of the clear ice layers of the ice block surface from the Little Reservation (Itcus et al., 2018). This could indicate a variability of the microbiome presence in different areas of the cave ice block. Interestingly, after a declining cell density in the ice layers formed during the last 5,000 years, microbial abundance displayed a spike in both the total and potentially active prokaryotic communities from 6,000 to 6,500-year-old ice, associated with a 2.5-fold increase in DOC concentration. Also, the higher microbial cell density in cave ice deposits older than 10,000 years appeared to be associated with the increased organic carbon content (Supplementary Figure S2A and Supplementary Table S1).

**FIGURE 8:**
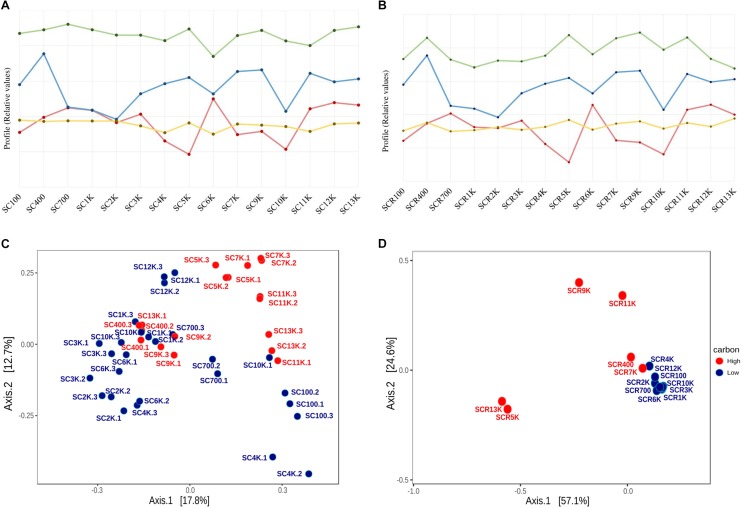
Impact of organic carbon concentrations on the abundance, diversity and phyla distribution on Scarisoara ice microbiome. Ice core profiles of DOC (blue; Supplementary Table S1), microbial cell density (red; Table 2), observed OTUs (green) and Shannon index (yellow; Table 3) values showing interrelated tendencies for **(A)** total (gDNA) and **(B)** potentially active (cDNA) communities; PCoA analysis of OTUs distribution based on the organic carbon content of ice layer of the **(C)** total community (gDNA) triplicate samples and **(D)** potentially active community (cDNA) samples.

Variation in the DOC content of the ice strata appeared to be correlated with the bacterial cell density, diversity and viability profiles across the 13,000-year-old cave ice block (Figure 8). The distribution of the cell density, observed OTUs, and Shannon index values of the total (Figure 8A) and potentially active (Figure 8B) communities displayed partially conserved profiles in relation to DOC concentrations. The cell density of ice microbiome (Figure 8A) paralleled the organic content profile across the ∼13,000-year-old ice block, except for the ice layers deposited during the 3,000–7,000 cal BP interval when microbial abundance and DOC content showed an opposite trend. Meanwhile, the cell density of the potentially active microbiome showed a comparable profile with that of DOC distribution along the corresponding ice strata (Figure 8B), in line with previously reports from Scarisoara ice formed during the last 900 years (Itcus et al., 2018). These results suggested the prevalence of ice depositional processes in modeling the structure of microbial communities accumulated in the supraglacial pond within Scarisoara cave prior to ice layer formation (Perşoiu et al., 2017), while more complex processes modeled the ice-entrapped prokaryotic community during the 3,000–7,000 cal BP interval.

The sharp increase of the geochemical components’ concentration in ice strata formed around 5,000 cal BP correlated with a lower ice-entrapped microbial abundance, and could be due to a higher input from the cave surface due to increased precipitation (Perşoiu et al., 2017). Meanwhile, the slightly higher concentrations of DOC and of several chemical compounds in the earlier part of the ice core could be related to enhanced erosion in the early Holocene, when forests were not fully established above the cave (Feurdean et al., 2014).

The observed OTUs and Shannon index profiles of the gDNA (Figure 8A) prokaryotic library also revealed a partial correlation throughout the ice core, with a similar trend for the 700–9,000-year-old ice interval, except for the 4,000-year-old ice sample. Meanwhile, ice layers formed during the last 700 years and from the late Holocene to the beginning of the Late Glacial period (9,000–13,000 cal BP) showed no correlation. In the case of the cDNA community (Figure 8B), diversity parameters and DOC values were highly correlated over the entire chronosequence (100–13,000-year-old ice samples) except for ice deposits formed 2,000–3,000 years ago and in the oldest ice layer (SCR13K). Thus, bacterial diversity in the Scarisoara ice cave appears to be strongly dependent on the depositional processes associated with ice formation from infiltration and dripping water from the cave surface (Feurdean et al., 2011; Perşoiu and Pazdur, 2011), in addition to direct precipitation falling through the large entrance shaft (Figure 1).

Principal coordinate analysis analysis of the OTUs variability of ice-contained prokaryotic communities based on the organic carbon content of ice core samples, indicated a very weak clustering (ANOSIM R: 0.114; *p* < 0.018; PERMANOVA F: 3.41; *R*^2^: 0.07; *p* < 0.001) of total microbial taxa from high and low carbon content strata, explaining 30.4% of the variability (Figure 8C). In the case of the putative active community, a stronger and significant correlation of these parameters occurred (ANOSIM R: 0.68; *p* < 0.001; PERMANOVA F: 7.80; *R*^2^: 0.37; *p* < 0.001), explaining 81.7% of the variation (Figure 8D). These results strongly support the critical role of the ice carbon content in shaping the bacterial community composition from the perennial ice block of Scarisoara cave. Moreover, the composition of the total prokaryotic community from the organic-rich strata SC9K and SC13K was similar to that from the low carbon content samples SC10K and SC12K (Figure 8C), implying more complex environmental factors for shaping the microbiome composition in old ice strata. The 4,000 and 7,000-year-old ice displayed comparable cDNA bacterial community structures despite their different carbon contents (Figure 8D), suggesting an important contribution of post-depositional processes occurring in the potentially active microbiome from these cave ice strata.

### Estimated Metabolic Activity Across the 13,000-Year-Old Cave Ice Block

Organic carbon variation through the ice core could be a result of post-depositional processes associated with the potentially active microbiome. Using the cell abundance of the viable community and DOC variations between ice strata, estimated metabolic rates of the microbiome entrenched in the cave ice chronosequence (Table 4) were calculated based on Price and Sowers (2004) approach. At the temperatures (∼0°C) that characterize Scarisoara Ice Cave (Perşoiu et al., 2011a), calculated metabolic rates varied between 2.32 × 10^-6^ g C (g C)^-1^ h^-1^ and 6.22 × 10^-5^ g C (g C)^-1^ h^-1^ corresponding to a maintenance metabolism (Morita, 1997) across the cave ice block. The apparent increases in microbial activity in 100, 400, 5,000, 7,000, and 9,000-year-old strata suggested a combination of growth and cell maintenance, according to the metabolic rates of microbial communities at 0°C from Lake Vostok subglacial ice (Karl et al., 1999), Antarctic Ace Lake (Franzmann et al., 1991), and deep seafloor marine sediments (D’Hondt et al., 2002) obtained by direct activity measurements. Comparable hypothetical metabolic rates in the (1.04–1.50) × 10^-5^ g C (g C)^-1^ h^-1^ range were calculated for the viable microbial community of the 900-year-old clear ice layer collected from border areas of Scarisoara ice block (Itcus et al., 2018) and the corresponding ice core layer from the central part of the ice block (this study). These estimated values suggested a correlation between the microbial cell density of the potentially active microbiome and the organic carbon content of ice strata, with little spatial variation across the ice block.

**Table 4:** Estimated metabolic rates of potentially active microbiome from Scarisoara ice core.

Samples	Metabolic rate [g C_DOC_ (g C_cells_)^-1^ h^-1^]	Type of metabolism
SCR100	6.44 × 10^–4^	Growth/maintenance
SCR400	3.90 × 10^–4^	Growth/maintenance
SCR700	7.59 × 10^–6^	Maintenance
SCR1K	1.04 × 10^–5^	Maintenance
SCR2K	4.70 × 10^–6^	Maintenance
SCR3K	8.11 × 10^–6^	Maintenance
SCR4K	6.22 × 10^–5^	Maintenance
SCR5K	3.88 × 10^–4^	Growth/maintenance
SCR6K	2.32 × 10^–6^	Maintenance
SCR7K	1.11 × 10^–4^	Growth/maintenance
SCR9K	1.34 × 10^–4^	Growth/maintenance
SCR10K	1.99 × 10^–5^	Maintenance
SCR11K	1.32 × 10^–5^	Maintenance
SCR12K	6.48 × 10^–6^	Maintenance
SCR13K	1.24 × 10^–5^	Maintenance


*Calculation of metabolic rates and type of metabolism assignment were performed according to Price and Sowers (2004), as indicated in Section “Materials and Methods.”*

The negative correlation between the viable cell density and DOC concentrations in 3,000–7,000-year-old ice strata (Figure 8) could be explained by enhanced post-depositional processes during this interval being associated with a more active cave ice microbial community adapted to sub-zero temperatures. This hypothesis was also supported by the clustering of bacterial taxa from SCR4K and SCR7K samples of low and high carbon content, respectively (Figure 8), in accordance with an enhanced estimated metabolic rate. The presence of various methanogenic species belonging to *Methanosphaerula* and *Methanosarcina* genera in the potentially active microbiome, and of Methanomicrobia phylotypes in the cave microbiome from 5,000 cal BP ice deposits could also explain the reduced organic carbon content, consistent with an increased metabolic rate. In addition, the calculated cell viability across the ice core appeared to be correlated with the organic carbon content of the ice layers (Supplementary Figure S6) but with a reduced coefficient of determination (*R*^2^ = 0.51), suggesting a combined effect of this parameter on both the depositional and post-depositional processes of the potentially active microbiome.

The putative active bacterial community found in perennial ice deposited in Scarisoara Ice Cave during the last ∼13,000 years was composed of both autotrophs and heterotrophs. This complex microbial composition suggested that this type of icy habitat was able to support various metabolic processes, shaping a unique microbiome. The carbon content of the ice strata appeared to be a major factor in determining the variability of microbial communities in the ice, in addition to playing an important role in depositional and post-depositional processes. This initial investigation of microbial communities entrapped in perennial cave ice since the Late Glacial through Holocene could help untangling the glacial microbiome response to climate variations. Moreover, the corroborated geochemical signature, cell abundance and diversity profiles of cave ice prokaryotic community along the ice core, in addition to stable isotope data characterizing the climate of the area (Perşoiu et al., 2017), suggested that a major environmental event could have occurred 4,500–5,000 years BP, constituting a starting point for identifying microbial climatic proxies and the possible formation of photosynthetic biofilms or microbial mat-like structures in the Scarisoara underground glacier.

## Author Contributions

VP and CP wrote the manuscript, with significant input from PL, AP, and CD. VP and AP participated to ice sampling. VP performed the sample filtration, DNA/RNA extraction, cDNA synthesis, and quantification experiments. GI and PL carried out the bioinformatics and statistical analyses. CM and AT conducted the geochemical analyses. AP contributed to depth-age model. CP performed the experimental design and coordinated the project. All authors participated in the data interpretation and revision of the manuscript.

## Conflict of Interest Statement

The authors declare that the research was conducted in the absence of any commercial or financial relationships that could be construed as a potential conflict of interest.

## References

[B1] Abraham W. P. Thomas S. (2015). Draft genome sequence of *Pseudomonas psychrophila* MTCC 12324, isolated from the Arctic at 79°N. *Genome Announc.* 3:e00578-15 10.1128/genomeA.00578-15 PMC4463518 26067953

[B2] Adams H. E. Crump B. C. Kling G. W. (2014). Metacommunity dynamics of bacteria in an arctic lake: the impact of species sorting and mass effects on bacterial production and biogeography. *Front. Microbiol.* 5:82. 10.3389/fmicb.2014.00082 24624127 PMC3940886

[B3] Alam S. I. Dixit A. Reddy G. S. N. Dube S. Palit M. Shivaji S. (2006). *Clostridium schirmacherense* sp. nov., an obligately anaerobic, proteolytic, psychrophilic bacterium isolated from lake sediment of Schirmacher Oasis, Antarctica. Int. J. Syst. Evol. Microbiol. 56(Pt 4), 715–720. 10.1099/ijs.0.63808-0 16585682

[B4] Alexeev S. V. Alexeeva L. P. Kononov A. M. (2016). Trace elements and rare earth elements in ground ice in kimberlites and sedimentary rocks of Western Yakutia. *Cold Reg. Sci. Technol.* 123 140–148. 10.1016/j.coldregions.2015.10.008

[B5] An L. Z. Chen Y. Xiang S. R. Shang T. C. Tian L. D. (2010). Differences in community composition of bacteria in four glaciers in western China. *Biogeosciences* 7 1937–1952. 10.5194/bg-7-1937-2010

[B6] Anesio A. M. Laybourn-Parry J. (2012). Glaciers and ice sheets as a biome. *Trends Ecol. Evol.* 27 219–225. 10.1016/j.tree.2011.09.012 22000675

[B7] Baqué M. de Vera J. P. Rettberg P. Billi D. (2013). The BOSS and BIOMEX space experiments on the EXPOSE-R2 mission: endurance of the desert cyanobacterium *Chroococcidiopsis* under simulated space vacuum, Martian atmosphere, UVC radiation and temperature extremes. *Acta Astronaut.* 91 180–186. 10.1016/j.actaastro.2013.05.015

[B8] Boyd E. S. Peters J. W. (2013). New insights into the evolutionary history of biological nitrogen fixation. *Front. Microbiol.* 4:201 10.3389/fmicb.2013.00201 PMC3733012 23935594

[B9] Brad T. Itcus C. Pascu M. D. Perşoiu A. Hillebrand-Voiculescu A. a Iancu L. (2018). Fungi in perennial ice from Scărişoara Ice Cave (Romania). *Sci. Rep.* 8:10096. 10.1038/s41598-018-28401-1 29973683 PMC6031636

[B10] Bundeleva I. A. Shirokova L. S. Pokrovsky O. S. Bénézeth P. Ménez B. Gérard E. (2014). Experimental modeling of calcium carbonate precipitation by cyanobacterium *Gloeocapsa* sp. *Chem. Geol.* 374–375, 44–60. 10.1016/j.chemgeo.2014.03.007

[B11] Campen R. Sowers T. Alley R. (2003). Evidence of microbial consortia metabolizing within a low-latitude mountain glacier. *Geology* 31 231–234.

[B12] Canfield D. E. Kristensen E. Thamdrup B. (2005). Aquatic geomicrobiology. *Adv. Mar. Biol.* 48 1–599. 15797449 10.1016/S0065-2881(05)48017-7

[B13] Caporaso J. G. Kuczynski J. Stombaugh J. Bittinger K. Bushman F. D. Costello E. K. (2010). QIIME allows analysis of high-throughput community sequencing data. *Nat. Methods* 7 335–336. 10.1038/nmeth.f.303 20383131 PMC3156573

[B14] Carpenter E. J. Lin S. Capone D. G. (2000). Bacterial activity in South Pole snow. *Appl. Environ. Microbiol.* 66 4514–4517. 10.1128/AEM.66.10.4514-4517.2000 11010907 PMC92333

[B15] Clausen H. B. Vrana K. Bo Hansen S. Larsen L. B. Baker J. Siggaard-Andersen M. L. (2006). “Continental ice body in Dobšiná Ice Cave (Slovakia). Part II. Results of chemical and isotopic study,” in *Proceedings of the 2nd International Workshop on Ice Caves* Vol. 3 Demänovská Dolina, 29–37.

[B16] Claussen M. Kubatzki C. Brovkin V. Ganopolski A. Hoelzmann P. Pachur H.-J. (1999). Simulation of an abrupt change in saharan vegetation in the mid-holocene. *Geophys. Res. Lett.* 26 2037–2040. 10.1029/1999gl900494

[B17] Connell L. Staudigel H. (2013). Fungal diversity in a dark oligotrophic volcanic ecosystem (DOVE) on Mount Erebus, Antarctica. *Biology* 2 798–809. 10.3390/biology2020798 24832809 PMC3960884

[B18] Deming J. W. (2002). Psychrophiles and Polar regions. *Curr. Opin. Microbiol.* 5 301–309. 10.1016/s1369-5274(02)00329-6 12057685

[B19] Dhariwal A. Chong J. Habib S. King I. Agellon L. B. Xia J. (2017). MicrobiomeAnalyst - a web-based tool for comprehensive statistical, visual and meta-analysis of microbiome data. *Nucleic Acids Res.* 45 W180–W188. 10.1093/nar/gkx295 28449106 PMC5570177

[B20] D’Hondt S. Rutherford S. Spivack A. J. (2002). Metabolic activity of subsurface life in deep-sea sediments. *Science* 295 2067–2070. 10.1126/science.1064878 11896277

[B21] Dieser M. Nocker A. Priscu J. C. Foreman C. M. (2010). Viable microbes in ice: application of molecular assays to McMurdo Dry Valley lake ice communities. *Antarct. Sci.* 22 470–476. 10.1017/s0954102010000404

[B22] Feurdean A. Perşoiu A. Pazdur A. Onac B. P. (2011). Evaluating the palaeoecological potential of pollen recovered from ice in caves: a case study from Scãrişoara Ice Cave, Romania. *Rev. Palaeobot. Palynol.* 165 1–10. 10.1016/j.revpalbo.2011.01.007

[B23] Feurdean A. Perşoiu A. Tanţãu I. Stevens T. Magyari E. K. Onac B. P. (2014). Climate variability and associated vegetation response throughout Central and Eastern Europe (CEE) between 8 and 60 kyrs ago. *Quat. Sci. Rev.* 106 206–224. 10.1016/j.quascirev.2014.06.003

[B24] Filippidou S. Wunderlin T. Junier T. Jeanneret N. Dorador C. Molina V. (2016). A combination of extreme environmental conditions favors the prevalence of endospore-forming Firmicutes. *Front. Microbiol.* 7:1707. 10.3389/fmicb.2016.01707 27857706 PMC5094177

[B25] Franzmann P. D. Roberts N. J. Mancuso C. A. Burton H. R. McMeekin T. A. (1991). Methane production in meromictic Ace Lake, Antarctica. *Hydrobiologia* 210 191–201. 10.1007/bf00034677

[B26] Ganzert L. Bajerski F. Mangelsdorf K. Lipski A. Wagner D. (2011). *Arthrobacter livingstonensis* sp. nov. and *Arthrobacter cryotolerans* sp. nov., salt-tolerant and psychrotolerant species from Antarctic soil. Int. J. Syst. Evol. Microbiol. 61(Pt 4), 979–984. 10.1099/ijs.0.021022-0 20511467

[B27] Grégori G. Denis M. Sgorbati S. Citterio S. (2018). Resolution of viable and membrane-compromised free bacteria in aquatic environments by flow cytometry. *Curr. Protoc. Cytom.* 85:e42. 10.1002/cpcy.42 29958333

[B28] Gunde-Cimerman N. Wagner D. Häggblom M. (2012). Polar and alpine microbiology. *FEMS Microbiol. Ecol.* 82 215–216. 10.1111/1574-6941.12004 23072484

[B29] Guo B. Liu Y. Gu Z. Shen L. Liu K. Wang N. (2016). *Massilia psychrophila* sp. nov., isolated from an ice core. Int. J. Syst. Evol. Microbiol. 66, 4088–4093 10.1099/ijsem.0.001315 27432318

[B30] Hansen A. A. Herbert R. A. Mikkelsen K. Jensen L. L. Kristoffersen T. Tiedje J. M. (2007). Viability, diversity and composition of the bacterial community in a high Arctic permafrost soil from Spitsbergen, Northern Norway. *Environ. Microbiol.* 9 2870–2884. 10.1111/j.1462-2920.2007.01403.x 17922769

[B31] Heylen K. Vanparys B. Peirsegaele F. Lebbe L. De Vos P. (2007). *Stenotrophomonas terrae* sp. nov. and *Stenotrophomonas humi* sp. nov., two nitrate-reducing bacteria isolated from soil. Int. J. Syst. Evol. Microbiol. 57(Pt 9), 2056–2061. 10.1099/ijs.0.65044-0 17766871

[B32] Hillebrand-Voiculescu A. Itcus C. Ardelean I. Pascu D. Perşoiu A. Rusu A. (2014). Searching for cold-adapted microorganisms in the underground glacier of Scarisoara ice cave, Romania. *Acta Carsol.* 43 319–329. 10.3986/ac.v43i2-3.604

[B33] Hillebrand-Voiculescu A. Rusu A. Itcus C. Perşoiu A. Brad T. Pascu M. D. (2013). 16S-rRNA gene clone library from recent ice stalagmites of Scarisoara cave. *Rom. J. Biochem.* 50 109–118.

[B34] Hiras J. Wu Y. W. Eichorst S. A. Simmons B. A. Singer S. W. (2015). Refining the phylum Chlorobi by resolving the phylogeny and metabolic potential of the representative of a deeply branching, uncultivated lineage. *ISME J.* 10 833–845. 10.1038/ismej.2015.158 26325358 PMC4796922

[B35] Holmlund P. Onac B. P. Hansson M. Holmgren K. Mörth M. Nyman M. (2005). Assessing the palaeoclimate potential of cave glaciers: the example of the Scarisoara Ice Cave (Romania). *Geogr. Ann.* 87 193–201. 10.1111/j.0435-3676.2005.00252.x

[B36] Hubbard J. D. (2017). *3D Cave and Ice Block Morphology from Integrated Geophysical Methods: A Case Study at Scãrişoara Ice Cave, Romania*. Available at: http://scholarcommons.usf.edu/etd/6712

[B37] Inoue K. Komagata K. (1976). Taxonomic study on obligately psychrophilic bacteria isolated from Antarctica. *J. Gen. Appl. Microbiol.* 22 165–176. 10.2323/jgam.22.165

[B38] Itcus C. Pascu M. D. Brad T. Perşoiu A. Purcarea C. (2016). Diversity of cultured bacteria from the perennial ice block of Scarisoara Ice Cave, Romania. *Int. J. Speleol.* 45 89–100. 10.5038/1827-806X.45.1.1948

[B39] Itcus C. Pascu M. D. Lavin P. Perşoiu A. Iancu L. Purcarea C. (2018). Bacterial and archaeal community structures in perennial cave ice. *Sci. Rep.* 8:15671. 10.1038/s41598-018-34106-2 30353134 PMC6199274

[B40] Karl D. M. Bird D. F. Bjorkman K. Houlihan T. Shackelford R. Tupas L. (1999). Microorganisms in the accreted ice of Lake Vostok, Antarctica. *Science* 286 2144–2147. 10.1126/science.286.5447.2144 10591643

[B41] Katayama T. Tanaka M. Moriizumi J. Nakamura T. Brouchkov A. Douglas T. A. (2007). Phylogenetic analysis of bacteria preserved in a permafrost ice wedge for 25 000 years. *Appl. Environ. Microbiol.* 73 2360–2363. 10.1128/AEM.01715-06 17293514 PMC1855676

[B42] Kern Z. Széles E. Horvatinčić N. Fórizs I. Bočić N. Nagy B. (2011). Geochemical investigations of the ice deposit of Vukušić Ice Cave, Velebit Mountain, Croatia. *Cryosphere* 5 485–494. 10.5194/tc-5-485-2011

[B43] Knowlton C. Veerapaneni R. D’Elia T. Rogers S. O. (2013). Microbial analyses of ancient ice core sections from Greenland and Antarctica. *Biology* 2 206–232. 10.3390/biology2010206 24832659 PMC4009855

[B44] Koci B. R. Kuivinen K. C. (1984). The PICO lightweight coring auger. *J. Glaciol.* 30 244–245. 10.1017/s0022143000006018

[B45] Lapanje A. Wimmersberger C. Furrer G. Brunner I. Frey B. (2012). Pattern of elemental release during the granite dissolution can be changed by aerobic heterotrophic bacterial strains isolated from Damma Glacier (central Alps) deglaciated granite sand. *Microb. Ecol.* 63 865–882. 10.1007/s00248-011-9976-7 22105516

[B46] Lauriol B. Prevost C. Lacelle D. (2006). The distribution of diatom flora in ice caves of the northern Yukon Territory, Canada: relationship to air circulation and freezing. *Int. J. Speleol.* 35 83–92. 10.5038/1827-806X.35.2.4

[B47] Leschine S. B. Canale-Parola E. (1989). Carbon cycling by cellulose-fermenting nitrogen-fixing bacteria. *Adv. Space Res.* 9 149–152. 10.1016/0273-1177(89)90039-2 17799733

[B48] Liu Q. Xin Y. H. Chen X. L. Liu H. C. Zhou Y. G. Chen W. X. (2018). *Cryobacterium aureum* sp. nov., a psychrophilic bacterium isolated from glacier ice collected from the ice tongue surface. Int. J. Syst. Evol. Microbiol. 68 1173–1176. 10.1099/ijsem.0.002647 29461184

[B49] Liu Y. Priscu J. C. Yao T. Vick-Majors T. J. Xu B. Jiao N. (2015). Bacterial responses to environmental change on the Tibetan Plateau over the past half century. *Environ. Microbiol.* 18 1930–1941. 10.1111/1462-2920.13115 26530871

[B50] Liu Y. Yao T. Jiao N. Kang S. Zeng Y. Liu X. (2009). Abundance and diversity of snow bacteria in two glaciers at the Tibetan Plateau. *Front. Earth Sci.* 3 80–90. 10.1007/s11707-009-0016-6 21468724

[B51] Lopatina A. Medvedeva S. Shmakov S. Logacheva M. D. Krylenkov V. Severinov K. (2016). Metagenomic analysis of bacterial communities of Antarctic surface snow. *Front. Microbiol.* 7:398. 10.3389/fmicb.2016.00398 27064693 PMC4814470

[B52] Maczulak A. (2011). “Clostridium,” in *Encyclopedia of Microbiology*, ed. Schmidt T. (New York, NY: Facts on File, Inc), 168–173.

[B53] Margesin R. Gander S. Zacke G. Gounot A. M. Schinner F. (2003). Hydrocarbon degradation and enzyme activities of cold-adapted bacteria and yeasts. *Extremophiles* 7 451–458. 10.1007/s00792-003-0347-2 12942349

[B54] Margesin R. Schumann P. Spröer C. Gounot A. (2004). *Arthrobacter psychrophenolicus* sp. nov., isolated from an alpine ice cave. Int. J. Syst. Evol. Microbiol. 54 2067–2072. 10.1099/ijs.0.63124-0 15545436

[B55] Mateos-Rivera A. Yde J. C. Wilson B. Finster K. W. Reigstad L. J. Øvreås L. (2016). The effect of temperature change on the microbial diversity and community structure along the chronosequence of the sub-arctic glacier forefield of Styggedalsbreen (Norway). *FEMS Microbiol. Ecol.* 92:fnw038. 10.1093/femsec/fiw038 26902803

[B56] Mayewski P. A. Meeker L. D. Twickler M. S. Whitlow S. Yang Q. Lyons W. B. (1997). Major features and forcing of high-latitude northern hemisphere atmospheric circulation using a 110,000-year-long glaciochemical series. *J. Geophys. Res. Oceans* 102 26,345–26,366. 10.1029/96jc03365

[B57] McCann C. M. Wade M. J. Gray N. D. Roberts J. A. Hubert C. R. J. Graham D. W. (2016). Microbial communities in a high arctic polar desert landscape. *Front. Microbiol.* 7:419. 10.3389/fmicb.2016.00419 27065980 PMC4814466

[B58] McMurdie P. J. Holmes S. (2013). phyloseq: an R Package for reproducible interactive analysis and graphics of microbiome census data. *PLoS One* 8:e61217. 10.1371/journal.pone.0061217 23630581 PMC3632530

[B59] Mercier C. Boyer F. Bonin A. Coissac E. (2013). “SUMATRA and SUMACLUST: fast and exact comparison and clustering of sequences,” in *Proceedings of the Abstract from the SeqBio 2013 Workshop*, Montpellier, 27–29.

[B60] Miteva V. Teacher C. Sowers T. Brenchley J. (2009). Comparison of the microbial diversity at different depths of the GISP2 Greenland ice core in relationship to deposition climates. *Environ. Microbiol.* 11 640–656. 10.1111/j.1462-2920.2008.01835.x 19278450

[B61] Mondini A. Donhauser J. Itcus C. Constantin M. Perşoiu A. Lavin P. (2019). High-throughput sequencing of fungal community diversity across the perennial ice block of Scarisoara ice cave. *Ann. Glaciol.* 59 134–146. 10.1017/aog.2019.6

[B62] Morita R. Y. (1997). *Bacteria in Oligotrophic Environments: Starvation-Survival Lifestyle*. New York, NY: Chapman & Hall.

[B63] Nash M. V. Anesio A. M. Barker G. Tranter M. Varliero G. Eloe-Fadrosh E. A. (2018). Metagenomic insights into diazotrophic communities across Arctic glacier forefields. *FEMS Microbiol. Ecol.* 94:fiy114. 10.1093/femsec/fiy114 29901729 PMC6054269

[B64] Onac B. P. Perşoiu A. Racovita G. Tamas T. Viehmann I. (2007). *Scărioara*. Cluj-Napoca: Studia, 84.

[B65] Paliy O. Shankar V. (2016). Application of multivariate statistical techniques in microbial ecology. *Mol. Ecol.* 25 1032–1057. 10.1111/mec.13536 26786791 PMC4769650

[B66] Perşoiu A. Onac B. P. Wynn J. G. Blaauw M. Ionita M. Hansson M. (2017). Holocene winter climate variability in Central and Eastern Europe. *Sci. Rep.* 7:1196. 10.1038/s41598-017-01397-w 28446780 PMC5430645

[B67] Perşoiu A. Onac B. P. Perşoiu I. (2011a). The interplay between air temperature and ice dynamics in Scărioara Ice Cave, Romania. *Acta Carsol.* 40 445–456. 10.3986/ac.v40i3.4

[B68] Perşoiu A. Onac B. P. Wynn J. G. Bojar A. V. Holmgren K. (2011b). Stable isotope behaviour during cave ice formation by water freezing in Scarisoara Ice Cave, Romania. *J. Geophys. Res. Atmos.* 116:D02111 10.1029/2010JD014477

[B69] Perşoiu A. Pazdur A. (2011). Ice genesis and its long-term mass balance and dynamics in Scarisoara Ice Cave, Romania. *Cryosphere* 5 45–53. 10.5194/tc-5-45-2011

[B70] Perşoiu I. Perşoiu A. (2018). Flood events in Transylvania during the medieval warm period and the little ice age. *Holocene* 29 85–96. 10.1177/0959683618804632

[B71] Pfennig N. Overmann J. (2015). “*Chlorobium*,” in *Bergey’s Manual of Systematics of Archaea and Bacteria*, ed. Whitman W. B. (Hoboken, NJ: Wiley), 1–9. 10.1002/9781118960608.gbm00374

[B72] Pop E. (1949). Bacterii nitrificante in pestera de la Scarisoara. *Bul. Stiint.* 1 901–907.

[B73] Popa R. Smith A. R. Popa R. Boone J. Fisk M. (2012). Olivine-respiring bacteria isolated from the rock-ice interface in a lava-tube cave, a Mars analog environment. *Astrobiology* 12 9–18. 10.1089/ast.2011.0639 22165996 PMC3264960

[B74] Price P. B. Sowers T. (2004). Temperature dependence of metabolic rates for microbial growth, maintenance, and survival. *Proc. Natl. Acad. Sci. U.S.A.* 101 4631–4636. 10.1073/pnas.0400522101 15070769 PMC384798

[B75] Priscu J. C. Adams E. E. Lyons W. B. Voytek M. A. Mogk D. W. Brown R. L. (1999). Geomicrobiology of subglacial ice above Lake Vostok, Antarctica. *Science* 286 2141–2144. 10.1126/science.286.5447.2141 10591642

[B76] Priscu J. C. Christner B. C. (2004). “Earth’s icy biosphere,” in *Microbial Biodiversity and Bioprospecting*, ed. Bull A. T. (Washington, DC: American Society for Microbiology Press), 130–145. 10.1128/9781555817770.ch13

[B77] Priscu J. C. Fritsen C. H. Adams E. E. Giovannoni S. J. Paerl H. W. McKay C. P. (1998). Perennial Antarctic lake ice: an oasis for life in a Polar desert. *Science* 280 2095–2098. 10.1126/science.280.5372.2095 9641910

[B78] Purcarea C. (2018). “Microbial life in ice caves,” in *Ice Caves*, eds Perçoiu A. Lauritzen S. E. (Atlanta, GA: Elsevier Inc), 173–187. 10.1016/b978-0-12-811739-2.00008-5

[B79] Pylro V. S. Roesch L. F. W. Morais D. K. Clark I. M. Hirsch P. R. Tótola M. R. (2014). Data analysis for 16S microbial profiling from different benchtop sequencing platforms. *J. Microbiol. Methods* 107 30–37. 10.1016/j.mimet.2014.08.018 25193439

[B80] Racovita E. G. Onac B. P. (2000). *Scărioara Glacier Cave: Monographic Study*. Cluj-Napoca: Carpatica Publishing Company.

[B81] Raven J. A. Kubler J. E. Beardall J. (2000). Put out the light, and then put out the light. *J. Mar. Biol. Assoc. U.K.* 80 1–25. 10.1017/s0025315499001526

[B82] Rivkina E. M. Friedmann E. I. McKay C. P. Gilichinsky D. A. (2000). Metabolic activity of permafrost bacteria below the freezing point. *Appl. Environ. Microbiol.* 66 3230–3233. 10.1128/aem.66.8.3230-3233.2000 10919774 PMC92138

[B83] Rognes T. Flouri T. Nichols B. Quince C. Mahé F. (2016). VSEARCH: a versatile open source tool for metagenomics. *PeerJ* 4:e2584. 10.7717/peerj.2584 27781170 PMC5075697

[B84] Santibáñez P. A. Maselli O. J. Greenwood M. C. Grieman M. M. Saltzman E. S. McConnell J. R. (2018). Prokaryotes in the WAIS Divide ice core reflect source and transport changes between Last Glacial Maximum and the early Holocene. *Glob. Change Biol.* 24 2182–2197. 10.1111/gcb.14042 29322639

[B85] Sattler B. Wille A. Waldhuber S. Sipiera P. Psenner R. (2002). “Various ice ecosystems in alpine and polar regions - an overview,” in *Proceedings of the First European Workshop on Exo-Astrobiology, 16–19 September 2002 Graz, Austria*, ed. Lacoste H. (Noordwijk: ESA Publications Division), 223–226.

[B86] Segata N. Izard J. Waldron L. Gevers D. Miropolsky L. Garrett W. S. (2011). Metagenomic biomarker discovery and explanation. *Genome Biol.* 12:R60. 10.1186/gb-2011-12-6-r60 21702898 PMC3218848

[B87] Sheik C. S. Stevenson E. I. Den Uyl PA Arendt C. A. Aciego S. M. Dick G. J. (2015). Microbial communities of the Lemon Creek Glacier show subtle structural variation yet stable phylogenetic composition over space and time. *Front. Microbiol.* 6:495. 10.3389/fmicb.2015.00495 26042114 PMC4438255

[B88] Shen L. Zhou Y. Liu H. Wang N. Jiao N. Xu B. (2015). *Massilia eurypsychrophila* sp. nov. a facultatively psychrophilic bacteria isolated from ice core. Int. J. Syst. Evol. Microbiol. 65 2124–2129. 10.1099/ijs.0.00022 25851590

[B89] Skidmore M. Anderson S. P. Sharp M. J. Foght J. M. Lanoil B. D. (2005). Comparison of microbial community composition in two subglacial environments reveals a possible role for microbes in chemical weathering processes. *Appl. Environ. Microbiol.* 71 6986–6997. 10.1128/AEM.71.11.6986-6997.2005 16269734 PMC1287656

[B90] Tabita F. R. Hanson T. E. Li H. Satagopan S. Singh J. Chan S. (2007). Function, structure, and evolution of the RubisCO-like proteins and their RubisCO homologs. *Microbiol. Mol. Biol. Rev.* 71 576–599. 10.1128/MMBR.00015-07 18063718 PMC2168653

[B91] Takahashi S. Tomita J. Nishioka K. Hisada T. Nishijima M. (2014). Development of a prokaryotic universal primer for simultaneous analysis of bacteria and archaea using Next-Generation Sequencing. *PLoS One* 9:e105592. 10.1371/journal.pone.0105592 25144201 PMC4140814

[B92] Tebo B. M. Davis R. E. Anitori R. P. Connell L. B. Schiffman P. Staudigel H. (2015). Microbial communities in dark oligotrophic volcanic ice cave ecosystems of Mt. Erebus, Antarctica. *Front. Microbiol.* 6:179 10.3389/fmicb.2015.00179 PMC4356161 25814983

[B93] Teehera K. B. Jungbluth S. P. Onac B. P. Acosta-Maeda T. E. Hellebrand E. Misra A. K. (2017). Cryogenic minerals in Hawaiian Lava Tubes: a geochemical and microbiological exploration. *Geomicrobiol. J.* 35 227–241. 10.1080/01490451.2017.1362079

[B94] Tveit A. Schwacke R. Svenning M. M. Urich T. (2012). Organic carbon transformations in high-Arctic peat soils: key functions and microorganisms. *ISME J.* 7 299–311. 10.1038/ismej.2012.99 22955232 PMC3554415

[B95] Walker V. K. Palmer G. R. Voordouw G. (2006). Freeze – thaw tolerance and clues to the winter survival of a soil community. *Appl. Environ. Microbiol.* 72 1784–1792. 10.1128/AEM.72.3.1784-1792.2006 16517623 PMC1393208

[B96] Whitman W. B. Coleman D. C. Wiebe W. J. (1998). Prokaryotes: the unseen majority. *Proc. Natl. Acad. Sci. U.S.A.* 95 6578–6583. 10.1073/pnas.95.12.6578 9618454 PMC33863

[B97] Wu Y. Tan L. Liu W. Wang B. Wang J. Cai Y. (2015). Profiling bacterial diversity in a limestone cave of the western Loess Plateau of China. *Front. Microbiol.* 6:244. 10.3389/fmicb.2015.00244 25870592 PMC4378288

[B98] Xu L. Wu Y. H. Jian S. L. Wang C. S. Wu M. Cheng L. (2016). *Pseudohongiella nitratireducens* sp. nov., isolated from seawater, and emended description of the genus *Pseudohongiella*. Int. J. Syst. Evol. Microbiol. 66 5155–5160. 10.1099/ijsem.0.001489 27612938

[B99] Zhang D. C. Schumann P. Liu H. C. Xin Y. H. Zhou Y. G. Schinner F. (2010). *Arthrobacter alpinus* sp. nov., a psychrophilic bacterium isolated from alpine soil. Int. J. Syst. Evol. Microbiol. 60(Pt 9), 2149–2153. 10.1099/ijs.0.017178-0 19880631

[B100] Zhang G. Niu F. Ma X. Liu W. Dong M. Feng H. (2007). Phylogenetic diversity of bacteria isolates from the Qinghai-Tibet Plateau permafrost region. *Can. J. Microbiol.* 53 1000–1010. 10.1139/w07-031 17898857

