# Supplementary material for: Total and Potentially Active Bacterial Communities Entrapped in a Late Glacial Through Holocene Ice Core From Scarisoara Ice Cave, Romania
Source: Front Microbiol. 2019 May 29;10:1193. doi: 10.3389/fmicb.2019.01193 (PMC6563852; doi:10.3389/fmicb.2019.01193)
Supplement: Supplementary file 1 [file Data_Sheet_1.PDF]

Supplementary Material

Total and potentially active bacterial communities entrapped in a Late Glacial through Holocene ice core from Scarisoara Ice Cave, Romania

Victoria I. Paun, Gonzalo Icaza[#], Paris Lavin[#], Constantin Marin, Alin Tudorache, Aurel Perşoiu, Cristina Dorador, Cristina Purcarea*

# Authors with equal contribution

*** Correspondence:** Cristina Purcarea, cristina.purcarea@ibiol.ro

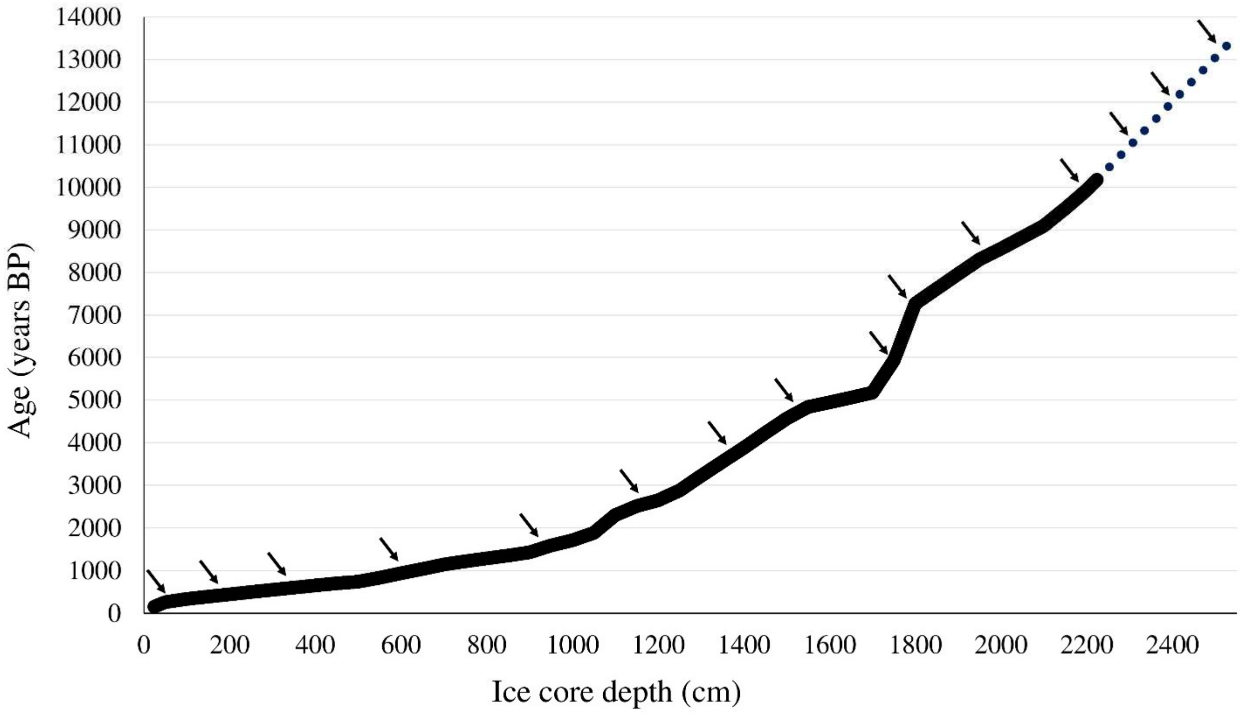

Supplementary Figure S1. Calculated ages of Scarisoara ice core. The age of ice core samples was determined by ^{14}C radiocarbon dating (line) and linear extrapolation for depth between 22.5 m and 25.33 m (dotted line), as indicated in Methods. The position of the 15 ice samples selected for gDNA and cDNA Illumina sequencing are indicated by arrows.

Supplementary Table S1. Geochemical characterization of Scarisoara cave ice core

Age (years)	BioSample	pH	DIC (µg g^{-1})	DOC (µg g^{-1})	TDS (µg g^{-1})	Na (ng g^{-1})	K (ng g^{-1})	Mg (ng g^{-1})	Ca (µg g^{-1})
92 ±25	SC(R)100	8.87±0.44	7.4±0.7	79.6±8.0	13.46	198.6±15.2	104.9±9.8	109.6±5.5	11.2±0.7
430 ±14	SC(R)400	8.59±0.43	3.6±0.4	590±59	19.09	406.4±31.0	344.1±32.1	136.9±508	8.7±0.6
475 ±17	-	n.a.	2.1±0.2	30.8±3.1	4.80	11.9±0.9	10.8±1.0	10.4±0.5	3.2±0.2
703 ±23	SC(R)700	7.50±0.38	5.4±0.5	18.4±1.8	21.71	622.1±47.5	2425±226	917.5±46.3	10.0±0.6
953 ±7	-	n.a.	2.8±0.3	30.5±3.1	6.54	244.5±18.6	346.0±32.3	172.8±8.7	4.0±0.3
1,124 ±31	SC(R)1K	9.22±0.46	3.9±0.4	15.1±1.5	7.55	76.5±5.8	229.8±21.5	138.1±7.0	5.4±0.3
1,671 ±36	SC(R)2K	7.39±0.37	1.5±0.1	8.3±0.8	2.28	22.2±1.7	3.7±0.3	16.6±0.8	1.5±0.1
2,671 ±36	SC(R)3K	9.59±0.48	4.0±0.4	43.9±4.4	8.29	188.1±14.3	201.1±18.8	237.6±12.0	6.0±0.4
3,937 ±66	SC(R)4K	7.03±0.35	2.7±0.3	84.9±8.5	5.08	71.9±5.5	25.1±2.3	33.2±1.7	3.6±0.2
4,683 ±23	-	n.a.	6.8±0.7	273±27	12.85	292.8±22.3	102.5±9.6	94.7±4.8	9.4±0.6
4,991 ±25	SC(R)5K	9.47±0.47	3.2±0.3	127±13	19.50	711.7±54.3	2653±248	2211±112	9.7±0.6
5,335 ±54	-	n.a.	3.4±0.3	17.1±1.7	6.34	92.5±7.1	75.2±7.0	62.2±3.1	5.1±0.3
6,159 ±177	SC(R)6K	9.22±0.46	4.0±0.4	43.5±4.3	5.84	154.6±11.8	102.3±9.6	86.4±4.4	4.5±0.3
7,124 ±143	-	n.a.	3.6±0.4	12.6±1.3	7.12	82.5±6.3	60.9±5.7	61.6±3.1	4.8±0.3
7,382 ±39	SC(R)7K	9.02±0.45	3.1±0.3	188±19	8.24	302.4±23.1	313.5±29.3	252.5±12.7	5.1±0.3
8,674 ±59	SC(R)9K	7.64±0.38	3.8±0.4	209±21	8.01	248.1±18.9	138.5±12.9	93.6±4.7	5.9±0.4
9,130 ±28	-	n.a.	3.5±0.3	253±25	7.64	183.6±14.0	99.7±9.3	93.9±4.7	5.3±0.3
10,002 ±126	SC(R)10K	6.91±0.35	4.4±0.4	14.0±1.4	9.19	149.1±11.4	158.5±14.8	99.6±5.0	7.0±0.5
11,102 ±114	SC(R)11K	9.15±0.46	4.3±0.4	167±17	12.84	306.0±23.3	77.0±7.2	61.5±3.1	6.2±0.4
12,007 ±89	SC(R)12K	8.90±0.45	4.3±0.4	95.7±9.6	10.21	254.2±19.4	377.9±35.3	298.5±15.1	7.1±0.5
13,098 ±29	-	n.a.	6.3±0.6	461±46	11.96	417.3±31.8	360.5±33.7	228.6±11.5	8.3±0.5
13,145 ±115	SC(R)13K	8.29±0.41	5.0±0.5	116±12	7.87	253.5±19.3	195.3±18.2	113.3±5.7	5.4±0.3

Age (years)	BioSample	Mn (ng g^{-1})	Fe (ng g^{-1})	B (ng g^{-1})	Si (ng g^{-1})	P (ng g^{-1})	SO_4 (µg g^{-1})	Cl (µg g^{-1})
92 ±25	SC(R)100	3.94±0.27	1.24±0.08	6.2±0.8	128.1±11.9	7.1±0.7	1.07±0.11	0.63±0.04
430 ±14	SC(R)400	1.09±0.07	b.d.l.	8.0±1.0	829.7±76.8	10.0±0.9	8.07±0.81	0.58±0.03
475 ±17	-	2.02±0.14	b.d.l.	9.6±1.2	32.1±3.0	7.9±0.7	0.62±0.06	0.90±0.05
703 ±23	SC(R)700	3.83±0.26	9.94±0.67	15.9±2.0	51.4±4.8	10.7±1.0	5.54±0.55	2.11±0.12
953 ±7	-	0.26±0.02	2.34±0.16	5.7±0.7	35.0±3.2	2.9±0.3	0.67±0.07	1.06±0.06
1,124 ±31	SC(R)1K	0.81±0.05	2.15±0.14	4.7±0.6	16.7±1.5	2.7±0.2	0.84±0.8	0.84±0.05
1,671 ±36	SC(R)2K	3.70±0.25	0.40±0.03	8.5±1.1	24.4±2.3	2.3±0.2	0.48±0.05	0.22±0.1
2,671 ±36	SC(R)3K	1.04±0.07	2.35±0.16	5.0±0.6	73.4±6.8	4.0±0.4	0.71±0.07	0.87±0.05
3,937 ±66	SC(R)4K	0.85±0.06	b.d.l.	5.7±0.7	152.5±14.1	3.5±0.3	0.81±0.08	0.38±0.02
4,683 ±23	-	3.61±0.24	b.d.l.	5.6±0.7	406.2±37.6	27.4±2.5	1.77±0.18	0.75±0.04
4,991 ±25	SC(R)5K	3.02±0.20	15.84±1.07	9.9±1.2	156.4±14.5	3.9±0.4	1.47±0.15	2.57±0.15
5,335 ±54	-	2.13±0.14	0.86±0.06	6.2±0.8	25.3±2.3	2.7±0.2	0.44±0.04	0.53±0.03
6,159 ±177	SC(R)6K	0.60±0.04	b.d.l.	12.0±1.5	51.9±4.8	8.6±0.8	0.53±0.05	0.39±0.02
7,124 ±143	-	1.68±0.11	1.73±0.12	7.6±0.9	56.7±5.2	10.7±1.0	1.41±0.14	0.63±0.04
7,382 ±39	SC(R)7K	2.44±0.16	3.93±0.26	7.6±0.9	226.1±20.9	5.8±0.5	1.09±0.11	0.94±0.05
8,674 ±59	SC(R)9K	1.61±0.11	1.88±0.13	6.4±0.8	294.8±27.3	5.3±0.5	0.77±0.8	0.55±0.03
9,130 ±28	-	1.41±0.10	b.d.l.	5.1±0.6	371.8±34.4	7.2±0.7	0.86±0.09	0.72±0.04
10,002 ±126	SC(R)10K	1.23±0.08	10.55±0.71	8.7±1.1	60.6±5.6	12.0±1.1	1.03±0.10	0.66±0.04
11,102 ±114	SC(R)11K	6.20±0.42	b.d.l.	5.4±0.7	366.8±34.0	10.9±1.0	4.98±0.50	0.83±0.05
12,007 ±89	SC(R)12K	9.68±0.62	9.09±0.61	6.5±0.8	231.6±21.4	3.6±0.3	0.79±0.08	1.13±0.07
13,098 ±29	-	3.91±0.26	b.d.l.	5.7±0.7	629.4±58.3	25.4±2.3	1.47±0.15	0.52±0.03
13,145 ±115	SC(R)13K	3.41±0.23	2.03±0.14	6.0±0.7	183.2±17.0	13.3±1.2	1.11±0.11	0.59±0.03

The pH and concentrations of total dissolved sediments (TDS), dissolved inorganic carbon (DIC), dissolved organic carbon (DOC), and elements of melted ice samples were measured as indicated in Methods. The age interval of each sample was calculated from ^{14}C radiocarbon analysis (Supplementary Figure S1), taking into consideration the depth interval of ice core fragments. b.l.d.: below detection limit (0.17 ng g^{-1} for Fe).

Supplementary Table S2. Unique cDNA OTUs not found in the gDNA libraries

**Sample**	**gDNA library** (total OTUs number)	**cDNA library** (unique OTUs number)	**Unique cDNA OTUs fraction** (%)
**SCR100**	74,400	2	0.0027
**SCR400**	114,613	531	0.4633
**SCR700**	74,955	2	0.0027
**SCR1K**	21,106	1	0.047
**SCR2K**	21,877	46	0.2103
**SCR3K**	42,837	35	0.0817
**SCR4K**	23,143	47	0.2031
**SCR5K**	69,860	157	0.2247
**SCR6K**	110,119	1	0.0009
**SCR7K**	133,215	377	0.2830
**SCR9K**	174,996	658	0.3760
**SCR10K**	165,030	5	0.0030
**SCR11K**	126,446	218	0.1724
**SCR12K**	24,777	125	0.5045
**SCR13K**	55,001	505	0.9182

Unique OTUs from the cDNA libraries of the ice core samples represented sequences not found in the corresponding gDNA library.

Supplementary Table S3. One-way analysis of variance (ANOSIM) and Tukey's multiple comparison tests of total and viable cell density from Scarisoara ice layers

ANOVA	*Source*	*SS*	*df*	*MS*	*F*	*Significance*
Total cell density	Between	3.91E+09	14	2.80E+08	13.49	< 0.0001
	Within	6.22E+08	30	2.07E+07		
	Total	4.54E+09	44			
Active cell density	Between	1.84E+09	14	1.31E+08	5.78	< 0.0001
	Within	6.80E+08	30	2.27E+07		
	Total	2.52E+09	44			

TUKEY	*Ice strata (age)*	*Mean. Diff.*	*q*	*P < 0.05*	*Summary*	*95% CI of diff*
Total cell density	100 vs 700	-16289	6.197	0.009051	**	-29985 to -2593
	100 vs 6K	-30178	11.480	4.32E-07	***	-43874 to -16481
	100 vs 11K	-15222	5.792	0.018680	*	-28919 to -1526
	100 vs 12K	-23400	8.903	5.33E-05	***	-37096 to -9704
	100 vs 13K	-19733	7.508	0.000776	***	-33430 to -6037
	400 vs 6K	-22000	8.370	0.000148	***	-35696 to -8304
	400 vs 12K	-15222	5.792	0.018680	*	-28919 to -1526
	700 vs 4K	15533	5.910	0.015160	*	1837 to 29230
	700 vs 5K	16711	6.358	0.006752	**	3015 to 30407
	700 vs 6K	-13889	5.284	0.044370	*	-27585 to -192.5
	700 vs 7K	14489	5.513	0.030260	*	792.5 to 28185
	700 vs 9K	13778	5.242	0.047560	*	81.39 to 27474
	700 vs 10K	16378	6.231	0.008512	**	2681 to 30074
	1K vs 5K	14067	5.352	0.039660	*	370.3 to 27763
	1K vs 6K	-16533	6.290	0.007641	**	-30230 to -2837
	1K vs 10K	13733	5.225	0.048900	*	36.95 to 27430
	2K vs 6K	-24756	9.419	1.99E-05	***	-38452 to -11059
	2K vs 12K	-17978	6.840	0.002758	**	-31674 to -4281
	2K vs 13K	-14311	5.445	0.033930	*	-28007 to -614.7
	3K vs 6K	-19844	7.550	0.000716	***	-33541 to -6148
	4K vs 6K	-29422	11.190	7.27E-07	***	-43119 to -15726
	4K vs 11K	-14467	5.504	0.030700	*	-28163 to -770.3
	4K vs 12K	-22644	8.615	9.25E-05	***	-36341 to -8948
	4K vs 13K	-18978	7.220	0.001343	**	-32674 to -5281
	5K vs 6K	-30600	11.640	3.24E-07	***	-44296 to -16904
	5K vs 11K	-15644	5.952	0.014070	*	-29341 to -1948
	5K vs 12K	-23822	9.064	3.92E-05	***	-37519 to -10126
	5K vs 13K	-20156	7.668	0.000571	***	-33852 to -6459
	6K vs 7K	28378	10.800	1.50E-06	***	14681 to 42074
	6K vs 9K	27667	10.530	2.48E-06	***	13970 to 41363
	6K vs 10K	30267	11.520	4.06E-07	***	16570 to 43963
	6K vs 11K	14956	5.690	0.022300	*	1259 to 28652
	7K vs 12K	-21600	8.218	0.000198	***	-35296 to -7904
	7K vs 13K	-17933	6.823	0.002847	**	-31630 to -4237
	9K vs 12K	-20889	7.947	0.000334	***	-34585 to -7193
	9K vs 13K	-17222	6.552	0.004717	**	-30919 to -3526
	10K vs 11K	-15311	5.825	0.017610	*	-29007 to -1615
	10K vs 12K	-23489	8.937	4.99E-05	***	-37185 to -9793
	10K vs 13K	-19822	7.542	0.000728	***	-33519 to -6126

Active cell density	100 vs 6K	-17289	6.288	0.007673	**	-31616 to -2961
	100 vs 12K	-17644	6.417	0.006054	**	-31972 to -3317
	400 vs 12K	-14400	5.237	0.047920	*	-28728 to -72.46
	1K vs 6K	-15289	5.561	0.027870	*	-29616 to -961.4
	1K vs 12K	-15644	5.690	0.022310	*	-29972 to -1317
	2K vs 6K	-15556	5.658	0.023590	*	-29883 to -1228
	2K vs 12K	-15911	5.787	0.018830	*	-30239 to -1584
	4K vs 6K	-18178	6.611	0.004228	**	-32505 to -3850
	4K vs 12K	-18533	6.741	0.003322	**	-32861 to -4206
	5K vs 6K	-19156	6.967	0.002171	**	-33483 to -4828
	5K vs 12K	-19511	7.096	0.001700	**	-33839 to -5184
	6K vs 7K	17800	6.474	0.005454	**	3472 to 32128
	6K vs 9K	18022	6.555	0.004697	**	3695 to 32350
	6K vs 10K	18844	6.854	0.002687	**	4517 to 33172
	7K vs 12K	-18156	6.603	0.004292	**	-32483 to -3828
	9K vs 12K	-18378	6.684	0.003692	**	-32705 to -4050
	10K vs 12K	-19200	6.983	0.002106	**	-33528 to -4872

Age: years BP, where K corresponds to 1000 years*; SS*: sum-of-squares; *df*: degrees of freedom; *MS*: mean squares; *F-value*: variation between sample means / variation within the samples; *q:* studentized range indicating the difference between the largest and smallest data points of the sample standard deviations; *Mean. Diff.*: mean difference; *CI*: confidence interval; *P*: probability value

A

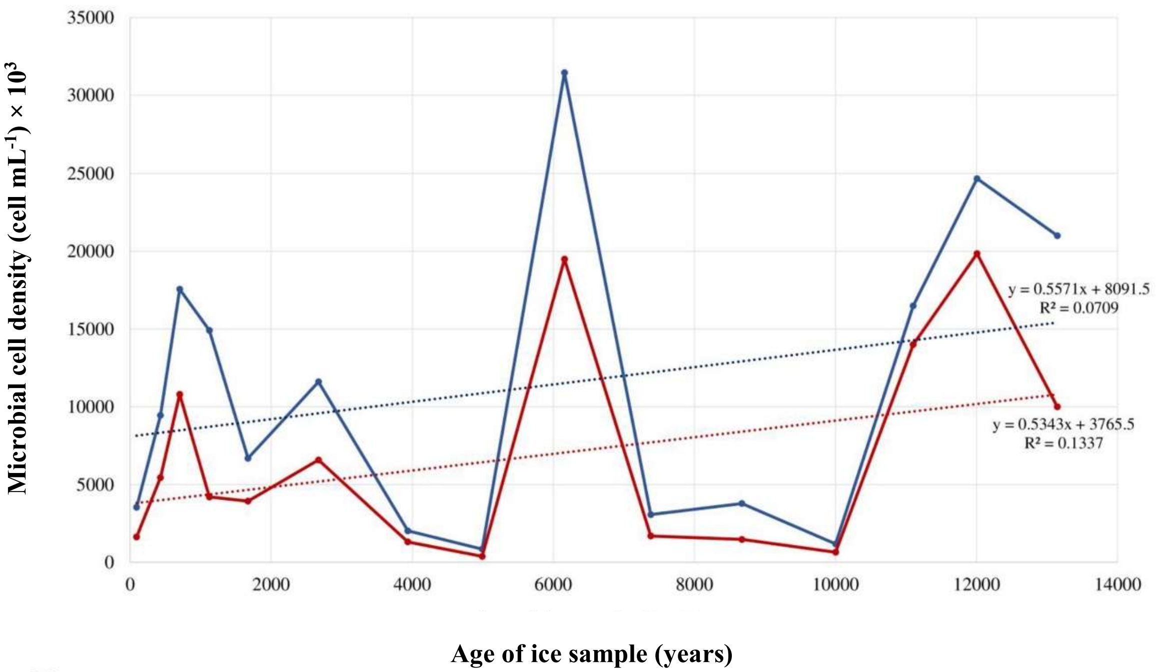

B

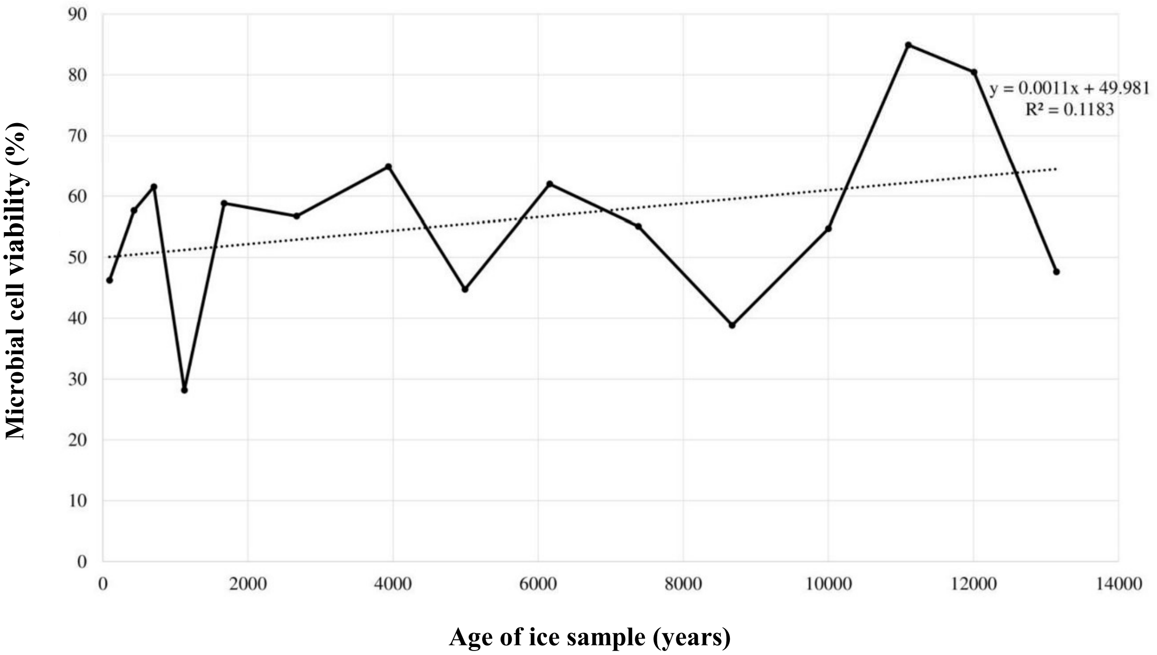

Supplementary Figure S2. Temporal variation of microbial cell density and viability across Scarisoara ice block. **(A)** Profile of microbial cell density from total (blue) and viable (red) communities (Table 2); **(B)** Microbial cell viability (Table 2) variation with the age of ice (black).

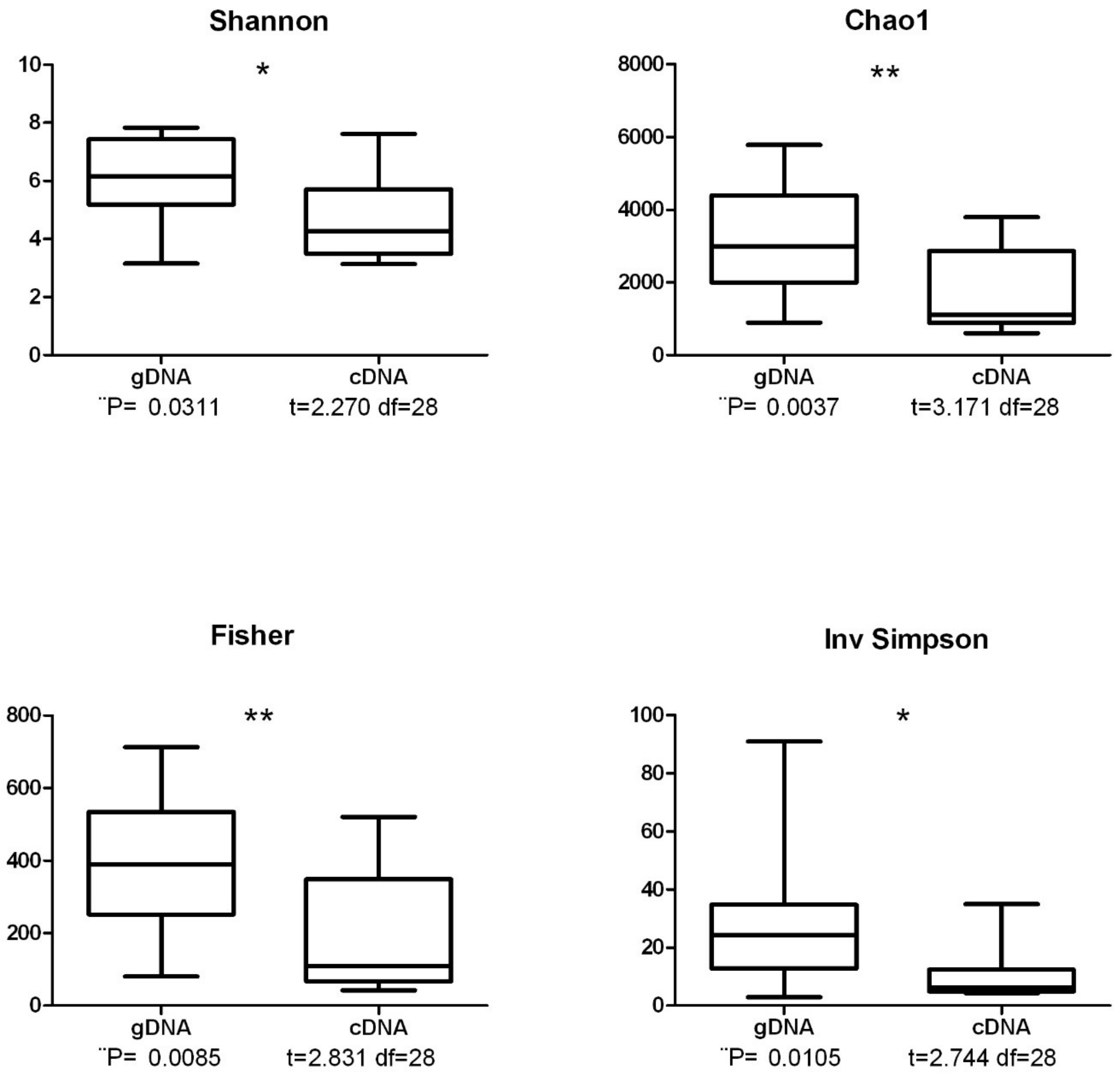

Supplementary Figure S3. T-test average values for diversity indices

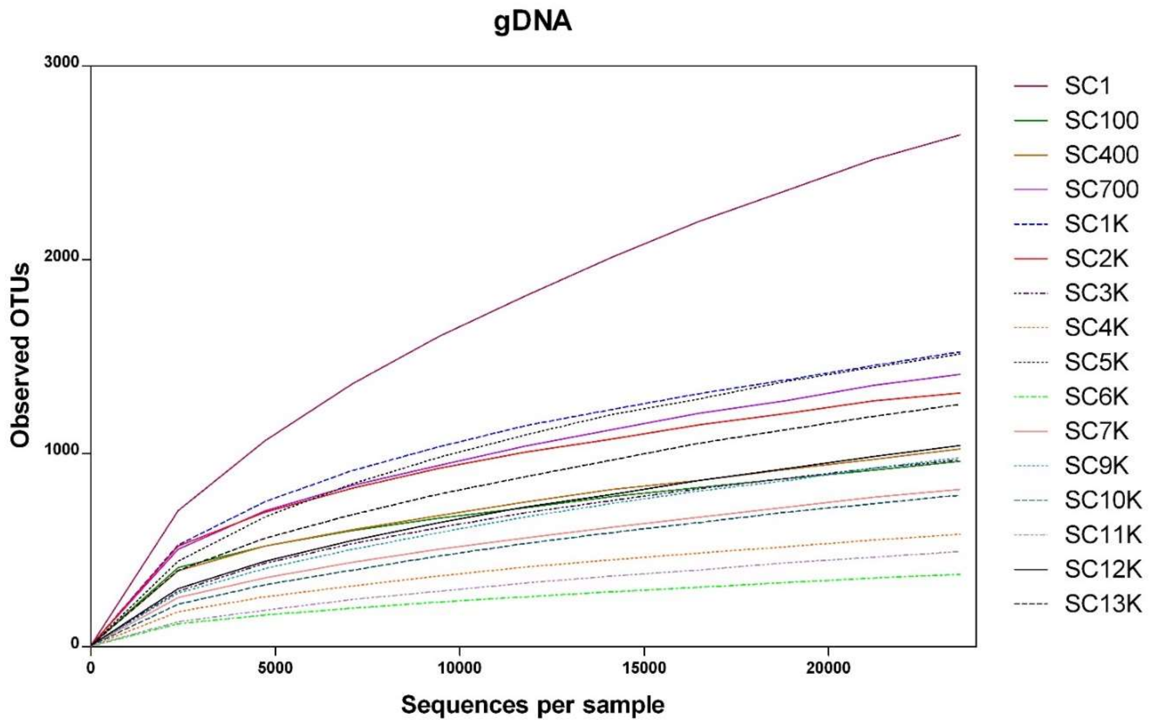

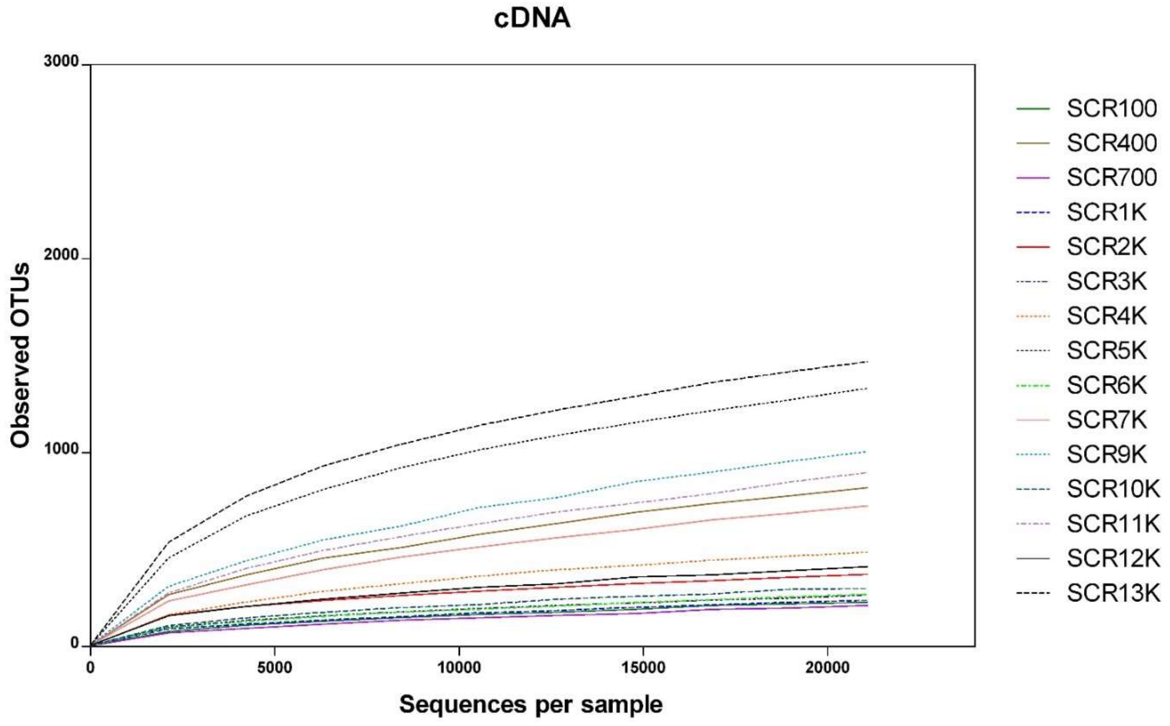

Supplementary Figure S4. Rarefaction curves

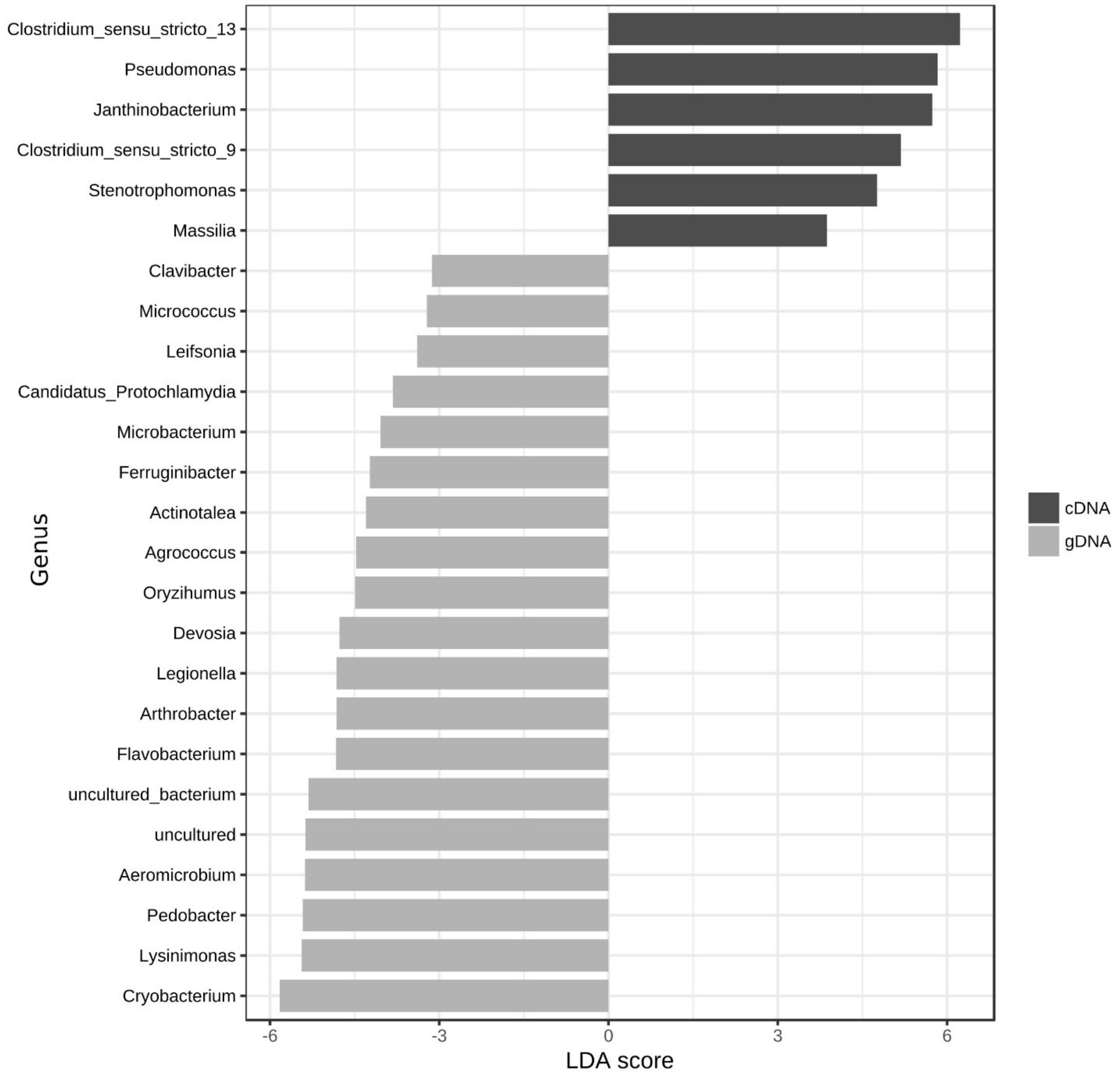

Supplementary Figure S5. Linear discriminant analysis effect size (LEfSe) of major bacterial taxa at genera level found in the total (grey) and potentially active (black) communities, based on the OTUs relative content from gDNA and cDNA 16S rRNA gene Illumina libraries, respectively. The bar plot indicates the LDA scores of the 25 most significant genera ($p < 0.05$).

Supplementary Table S4. Relative abundance of archaeal taxa across the ice block

Archaea taxonomy	gDNA														
	SC100	SC400	SC700	SC1K	SC2K	SC3K	SC4K	SC5K	SC6K	SC7K	SC9K	SC10K	SC11K	SC12K	SC13K
Bathyarchaeota; uncultured archaeon	0.00302	0	0	0	0	0	0	0	0	0	0	0	0	0	0
Euryarchaeota; Methanomicrobia; Methanomicrobiales, Methanoregulaceae; Methanosphaerula	0.0042	0.017	0	0	0	0.0030	0	0.00355	0	0.006281	0.002075	0	0	0.000209	0
Euryarchaeota; Methanomicrobia; Methanosarcinales; Methanosarcinaceae; Methanosarcina	0.02180	0	0.00145	0	0	0	0	0.00548	0	0.00288	0	0	0	0.001044	0
Euryarchaeota; Methanomicrobia; Methanosarcinales; Methanosarcinaceae; Methanosarcina	0	0	0	0	0	0	0	0.000968	0	0	0	0	0	0	0
Euryarchaeota; Thermoplasmata; Thermoplasmatales; Marine group II; uncultured archaeon	0	0.02140	0	0	0	0	0	0	0	0	0	0	0	0	0
Euryarchaeota; Thermoplasmata; Thermoplasmatales; Terrestrial Miscellaneous Gp (TMEG); uncultured	0	0.00165	0	0	0	0	0	0.00323	0	0	0	0	0	0	0
Archaea taxonomy	cDNA														
	SCR100	SCR400	SCR700	SCR1K	SCR2K	SCR3K	SCR4K	SCR5K	SCR6K	SCR7K	SCR9K	SCR10K	SCR11K	SCR12K	SCR13K
Bathyarchaeota; uncultured archaeon	0	0	0	0	0	0	0	0	0	0	0	0	0	0	0
Euryarchaeota; Methanomicrobia; Methanomicrobiales, Methanoregulaceae; Methanosphaerula	0	0	0	0	0	0	0	0.04437	0	0	0	0	0	0	0
Euryarchaeota; Methanomicrobia; Methanosarcinales; Methanosarcinaceae; Methanosarcina	0	0	0	0	0.1417	0	0	0.29058	0	0	0	0	0	0	0.00182
Euryarchaeota; Methanomicrobia; Methanosarcinales; Methanosarcinaceae; Methanosarcina	0	0	0	0	0	0	0	0.00286	0	0	0	0	0	0	0
Euryarchaeota; Thermoplasmata; Thermoplasmatales; Marine group II; uncultured archaeon	0	0	0	0	0	0	0	0	0	0	0	0	0	0	0
Euryarchaeota; Thermoplasmata; Thermoplasmatales; Terrestrial Miscellaneous Gp(TMEG); uncultured	0	0	0	0	0	0	0	0	0	0	0	0	0	0	0

Supplementary Table S5. Most abundant bacterial taxa from the putative active (cDNA) microbial community of Scarisoara ice block

OTUs	Taxonomic rank	Closest relative (GenBank)	Identity (%)	Origin
Otu00972	Firmicutes, Clostridia, Clostridiales, Clostridiaceae 1, Clostridium sensu stricto 13, uncultured bacterium	Uncultured *Clostridium sp.* (MF360138.1)	100	cattle rumen
Otu02063	Proteobacteria, Gammaproteobacteria, Pseudomonadales, Pseudomonadaceae, Pseudomonas, Other	Uncultured bacterium (FJ934538.1)	97	maple sap
Otu01713	Proteobacteria, Betaproteobacteria, Burkholderiales, Oxalobacteraceae, Janthinobacterium, Other	*Janthinobacterium sp.* (FJ889626.1)	95	soil, Norway
Otu01710	Proteobacteria, Betaproteobacteria, Burkholderiales, Oxalobacteraceae, Janthinobacterium, Ambiguous_taxa	*Janthinobacterium lividum* (MH929893.1)	95	tundra soil
Otu00981	Firmicutes, Clostridia, Clostridiales, Clostridiaceae 1, Clostridium sensu stricto 9, Other	Uncultured *Clostridium sp.* (JX505229.1)	94	soil
Otu02060	Proteobacteria, Gammaproteobacteria, Pseudomonadales, Pseudomonadaceae, Pseudomonas, uncultured bacterium	Uncultured bacterium (FN813929.1)	98	*Lactuca sativa* (cv. Saladin), phyllosphere
Otu02052	Proteobacteria, Gammaproteobacteria, Pseudomonadales, Pseudomonadaceae, Pseudomonas, *Pseudomonas fragi*	*Pseudomonas fragi* (KX186947.2)	95	beef meat
Otu02114	Proteobacteria, Gammaproteobacteria, Xanthomonadales, Xanthomonadaceae, Stenotrophomonas, Other	*Stenotrophomonas sp.* (AF170732.1)	95	Antarctic quartz stone
Otu00985	Firmicutes, Clostridia, Clostridiales, Clostridiaceae 1, Other, Other	*Clostridium putrefaciens* (NR_113324.1)	94	spoiled ham
Otu02048	Proteobacteria, Gammaproteobacteria, Pseudomonadales, Pseudomonadaceae, Pseudomonas, Ambiguous_taxa	*Pseudomonas fluorescens* (MH699401.1)	99	soil samples from rice fields
Otu00971	Firmicutes, Clostridia, Clostridiales, Clostridiaceae 1, Clostridium sensu stricto 13, uncultured *Clostridium sp.*	Uncultured *Clostridium sp.* (MF360138.1)	99	cattle rumen
Otu01720	Proteobacteria, Betaproteobacteria, Burkholderiales, Oxalobacteraceae, Massilia, Other	Uncultured bacterium (KC606946.1)	96	groundwater
Otu02051	Proteobacteria, Gammaproteobacteria, Pseudomonadales, Pseudomonadaceae, Pseudomonas, *Pseudomonas fluorescens*	Uncultured Pseudomonadaceae (JF733317.1)	91	*Simulium dixiense* (black fly)

Otu02055	Proteobacteria, Gammaproteobacteria, Pseudomonadales, Pseudomonadaceae, Pseudomonas, *Pseudomonas putida*	*Pseudomonas putida* (KT714143.1)	93	soil and water habitats
Otu00899	Firmicutes, Bacilli, Lactobacillales, Carnobacteriaceae, Carnobacterium, *Carnobacterium maltaromaticum*	Uncultured bacterium (EU777662.1)	97	Polar bear feces
Otu02054	Proteobacteria, Gammaproteobacteria, Pseudomonadales, Pseudomonadaceae, Pseudomonas, *Pseudomonas psychrophila*	*Pseudoalteromonas undina* (LS482978.1)	97	Arctic fjord water
Otu02110	Proteobacteria, Gammaproteobacteria, Xanthomonadales, Xanthomonadaceae, Stenotrophomonas, Ambiguous_taxa	*Stenotrophomonas sp.* (KC128832.1)	97	soil from Kashmir Cave Buner
Otu02151	Saccharibacteria, Other, Other, Other, Other, Other	*Candidatus Saccharibacteria* (CP025011.1)	93	rhizosphere soil
Otu00224	Actinobacteria, Actinobacteria, Micrococcales, Microbacteriaceae, Cryobacterium, Other	*Cryobacterium luteum* (MG973010.1)	95	cryoconite sediment
Otu02150	Saccharibacteria, uncultured bacterium, uncultured bacterium, uncultured bacterium, uncultured bacterium, uncultured bacterium	uncultured *Candidatus Saccharibacteria* (MF042893.1)	96	groundwater
Otu00032	Acidobacteria, Holophagae, Subgroup 7, Other, Other, Other	Uncultured bacterium (FQ658985.1)	99	PAH-contaminated soil
Otu00267	Actinobacteria, Actinobacteria, Micrococcales, Microbacteriaceae, Other, Other	*Microbacteriaceae bacterium* (KC478080.1)	96	Antarctic lakes
Otu00221	Actinobacteria, Actinobacteria, Micrococcales, Microbacteriaceae, Cryobacterium, Ambiguous_taxa	*Cryobacterium levicorallinum* (MH482270.1)	92	Arctic soil

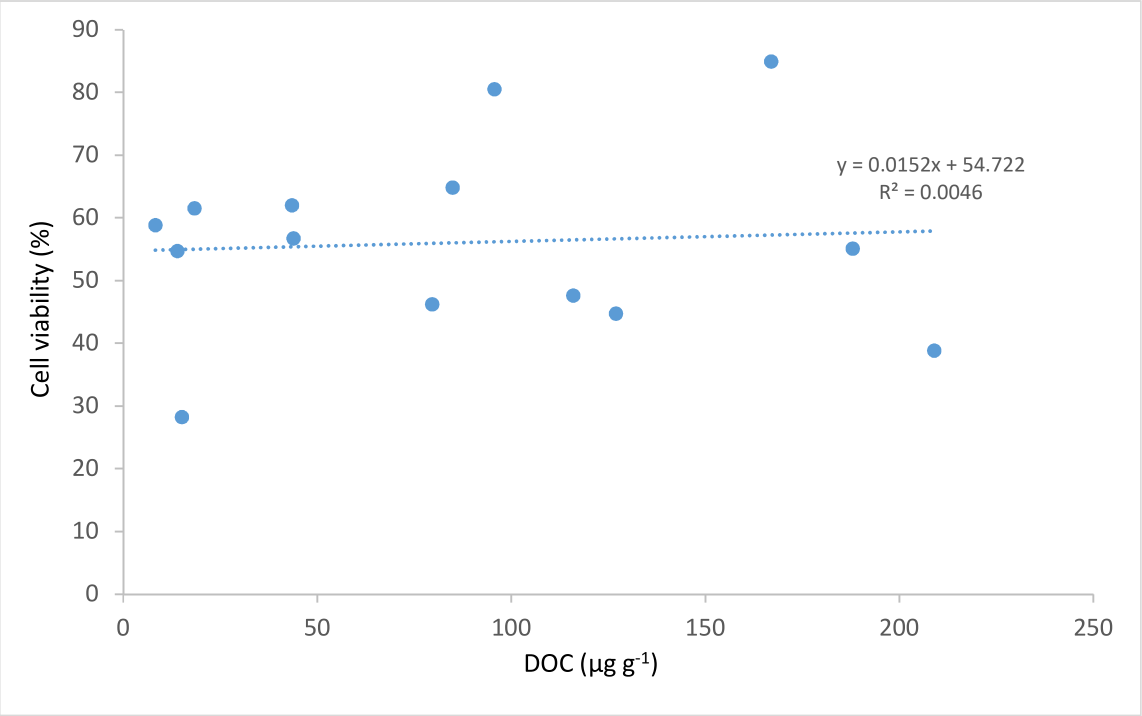

Supplementary Figure S6. Cell viability variation with the dissolved organic carbon (DOC) concentrations of Scarisoara cave ice strata
